# Modification of Seurat v4 for the Development of a Phase Assignment Tool Able to Distinguish between G2 and Mitotic Cells

**DOI:** 10.3390/ijms25094589

**Published:** 2024-04-23

**Authors:** Steven Watson, Harry Porter, Ian Sudbery, Ruth Thompson

**Affiliations:** 1 School of Medicine and Population Health, University of Sheffield, Sheffield S10 2TN, UK; 2 School of Medicine, University of Nottingham, Nottingham NG5 1PB, UK; 3 School of Biosciences, University of Sheffield, Sheffield S10 2TN, UK; 4 Sheffield Institute for Nucleic Acid Research (SInFoNiA), Sheffield S10 2TN, UK

**Keywords:** mitosis, bioinformatics, cell cycle, phase assignment, RNA sequencing

## Abstract

Single-cell RNA sequencing (scRNAseq) is a rapidly advancing field enabling the characterisation of heterogeneous gene expression profiles within a population. The cell cycle phase is a major contributor to gene expression variance between cells and computational analysis tools have been developed to assign cell cycle phases to cells within scRNAseq datasets. Whilst these tools can be extremely useful, all have the drawback that they classify cells as only G1, S or G2/M. Existing discrete cell phase assignment tools are unable to differentiate between G2 and M and continuous-phase-assignment tools are unable to identify a region corresponding specifically to mitosis in a pseudo-timeline for continuous assignment along the cell cycle. In this study, bulk RNA sequencing was used to identify differentially expressed genes between mitotic and interphase cells isolated based on phospho-histone H3 expression using fluorescence-activated cell sorting. These gene lists were used to develop a methodology which can distinguish G2 and M phase cells in scRNAseq datasets. The phase assignment tools present in Seurat were modified to allow for cell cycle phase assignment of all stages of the cell cycle to identify a mitotic-specific cell population.

## 1. Introduction

Single-cell RNA sequencing (scRNAseq) is a rapidly advancing technology allowing researchers to assay gene expression at a higher resolution than conventional bulk RNA sequencing. This technique has facilitated the characterisation of cellular heterogeneity in complex tissues leading to the discovery of novel cell populations. However, these tissues frequently contain cycling cells and the cell cycle phase is a major factor driving gene expression variance between cells. This can impact the ability of researchers to define whether two clusters identified in dimensionality reduction plots are two distinct cell types or the same cell type in a different phase of the cell cycle. To address this, many scRNAseq toolkits apply methodologies to assign the cell cycle phase to individual cells in scRNAseq datasets.

The cell cycle has been recognised as a major contributor to gene expression variance between cells [1,2,3], leading to the development of various cell cycle computational analysis tools in order to overcome this, including Oscope [4], Peco [5], reCAT [6], Cyclone [7] and Seurat [8,9]. Whilst these tools can be extremely useful in the analysis of scRNAseq data for the study of cell cycle-specific effects, all of these tools have the drawback that they classify cells as G1, S or G2/M and are unable to differentiate between G2 and M.

Due to the highly condensed nature of mitotic chromatin, it has previously been assumed that transcription is repressed in mitosis [10,11,12]. This assumption remained in place for many years due to technical limitations; however, in the last 10 years, there have been significant advances. In 2017 it was proposed that transcription is maintained at a low level throughout transcription followed by a “wave” of transcription towards the end of mitosis to allow for mitotic exit [13]. Ongoing transcription in mitosis has been observed at centromeres [14] and other chromosome-fragile sites [15], and whilst telomeres are thought to be largely transcriptionally silent in mitosis, in cells depending on the alternative lengthening of telomeres (ALT) pathway for telomere maintenance, telomere-repeat-containing RNA (TERRA) remains associated with telomeres in G2/M [16].

The objective of this research was to develop a tool which could accurately identify mitotic cells from a grouped G2/M population using the scRNAseq profile of the cell. We modified an existing cell cycle phase assignment tool (Seurat) so it was able to assign G2 and M based on gene lists developed by ourselves. To develop the gene lists, we used fluorescent-activated cell sorting to separate interphase and mitotic cells prior to RNAseq analysis for the generation of a gene list of mitotically upregulated genes.

## 2. Results

**Figure 1:**
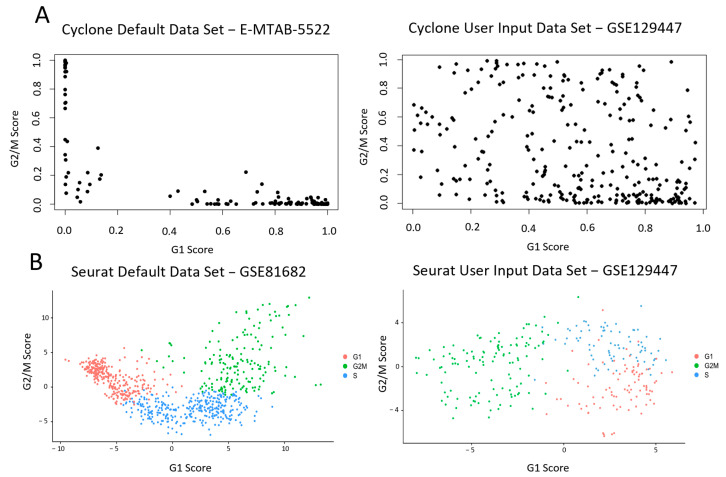
Finding a phase sorting code base which could be modified to analyse and detect mitotic cells. Both Cyclone and Seurat were tested for their comparative ability to determine and assign cell phases. Default datasets from each system and externally directed input datasets were tested. The effectiveness of phase assignment into G1, S and G2/M was tested in both systems. (**A**) Cyclone, a subfunction of the scran package, was tested for its effectiveness in phase assignment. Please see https://bioconductor.org/packages/release/bioc/html/scran.html (accessed on 21 October 2022) for further details. The left figure was generated from testing with the Cyclone default E-MTAB-5522 dataset. The right figure was generated from testing with the user-directed input GSE129447 dataset. (**B**) Seurat, specifically its Cell-Cycle Scoring functionality, was tested for its effectiveness in phase assignment. Please see https://satijalab.org/seurat/ (accessed on 21 October 2022) for further details. The left figure was generated from testing with the Seurat Cell-Cycle Scoring default GSE81682 dataset. The right figure was generated from testing with the user-directed input GSE129447 dataset.

We first assessed available online cell cycle phase assignment tools for their ability to assign cell cycle phases to a selected external dataset as compared with the dataset used in the development of the tool. After careful selection, we chose GSE129447 as it was a large, publicly available dataset from an early passage human-derived cell line with robust quality controls [17]. Here we show cell cycle phase assignment using two available tools, Cyclone [7] and Seurat [8], using both the datasets used in the development of each tool as controls and our selected dataset as a test. We found that while Cyclone [7] successfully clustered cells from the provided test dataset, no obvious clustering of the independent dataset was observed (Figure 1A). Seurat, however, successfully clustered both the independent dataset and the default dataset into discrete cell cycle phases (Figure 1B).

**Figure 2:**
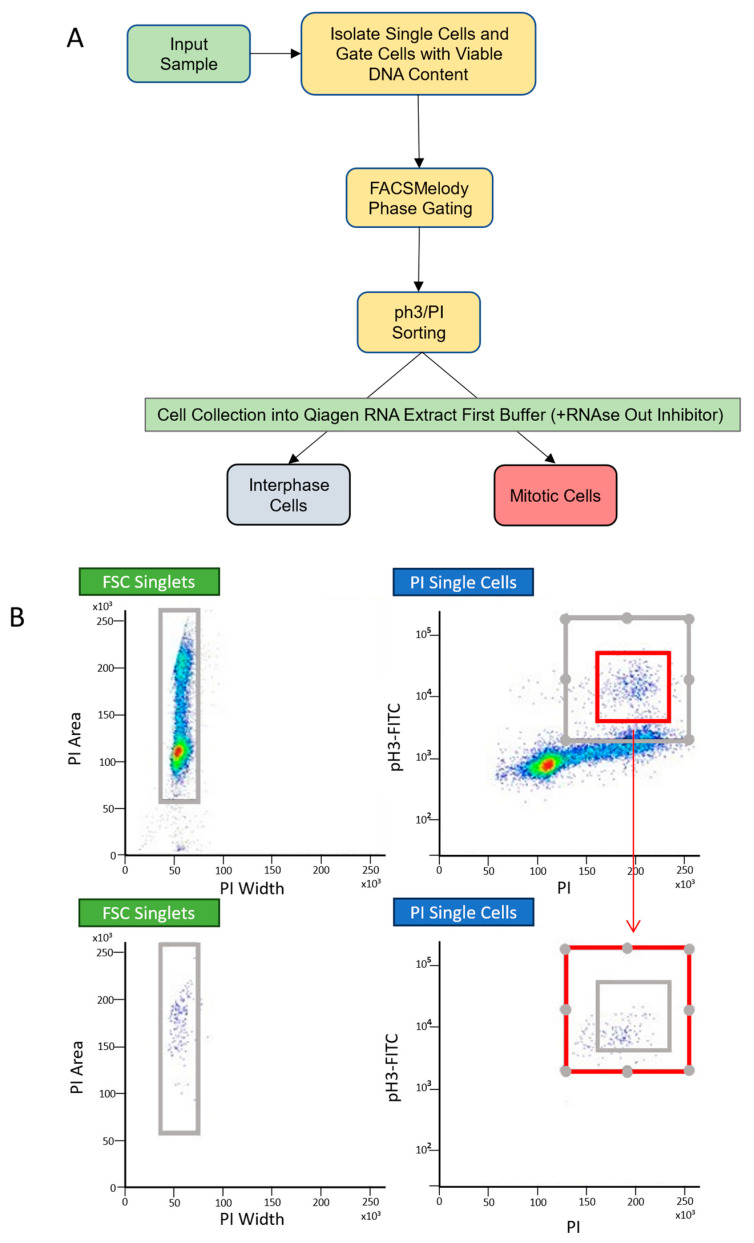
Separation of interphase and mitotic cells. (**A**) Workflow for separation of mitotic and interphase cells using FACSMelody. Cells were sorted based on phospho-histone H3- FITC staining. (**B**) HeLa cells were stained for pH3- FITC and PI and sorted using FACSMelody based on FITC staining. Representative plots show the separation of the phospho-histone H3-positive population. The red box shows pH3 expressing the 4N population. The lower plot shows this population only following the sort. The heatmap denotes cell density.

Based on the ability of Seurat to group an external dataset into distinct clusters (Figure 1B) we decided to proceed by modifying Seurat in order to identify mitotic cells. The cell cycle phase prediction methodology of Seurat involves assigning a score to each cell in a dataset based on gene expression lists for each cell cycle phase. We reasoned that this could be adapted to sort mitotic cells if a gene list specific to mitotic cells was provided. The default gene lists within the Seurat code separate G1, S and G2/M cell populations. Thus, a list of genes upregulated in mitotic cells was required. To generate this list, we sorted cells into mitotic and interphase fractions based on the expression of the mitotic marker histone H3 phosphorylated at Serine 10 (pH3) using fluorescence-activated flow cytometry [18] (Figure 2). This is a well-established method for the identification of mitotic cells by flow cytometry. To control for the possibility of pH3 expression in pre-replicated cells, only pH3 cells with >4N DNA content were sorted in the mitotic fraction. As the protocol for pH3 staining requires cells to be fixed, steps were taken to ensure good RNA quality and all our RNA samples had the RNA integrity tested and only sent for sequencing if the RIN was >8. We were able to separate the pH3-expressing population from the non-pH3-expressing population (Figure 2B).

**Figure 3:**
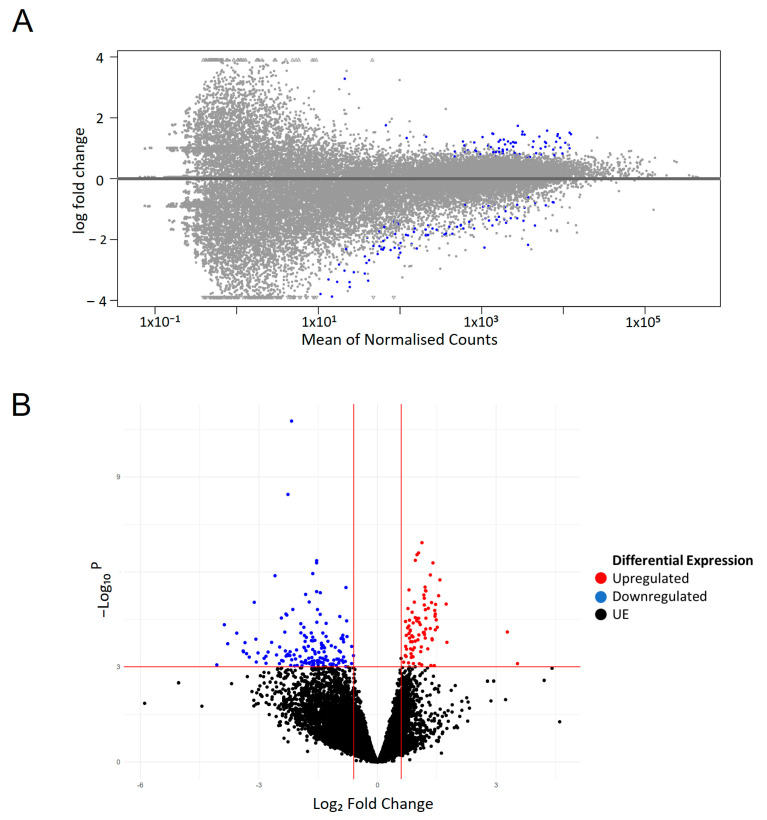
Differential gene expression testing via Deseq2. (**A**) MA plot of RNAseq data. Scatter plot of log2 fold changes versus the mean of normalised counts. Each dot represents a singular gene across all present. Blue dots represent differentially expressed genes under the preset padj < 0.05 threshold. Grey dots are not statistically significant. Blue dots are differentially expressed genes with a positive log fold change indicative of mitotic-related genes. The differentially expressed genes (blue points) with a negative log fold change are indicative of interphase-related genes. Data were plotted in R (https://www.r-project.org/ accessed 21 October 2022) using plotMA from deseq2 dds values regressing out the effect of treatment and replicate. (**B**) Differentially expressed genes in RNAseq volcano plot. Red data points represent upregulated mitotic weighted genes, and blue points represent downregulated interphase weighted genes. log2 Fold change of ±0.58 and padj value of ≤0.05. The volcano plot was created using the count matrix input and graphed via ggplot2. The X-axis is log2 fold change, y-axis is statistical significance −log10 (adjusted *p*-value).

Following cell sorting, RNA was extracted from four biological replicates of the sorted mitotic and interphase cells and following RIN analysis was sent out for bulk RNA sequencing. To minimise the possibility of genetic drift from the cells being maintained in culture, cells of the same frozen stock were thawed exactly 14 days prior to each sort and RNA extraction. RNAseq analysis of the eight samples (four interphase and four mitosis) revealed clear differentially expressed gene profiles between the mitotic and interphase populations (Figure 3A). At a standard threshold for differential expression (adjusted *p*-value < 0.05), we identified 76 genes preferentially expressed in interphase and 86 preferentially expressed in mitosis. To identify a high-confidence set of phase-specific genes, we used a more restrictive adjusted *p*-value cut-off of ≤0.001 (Figure 3B). The gene lists shown in Table 1 are the final lists used in our Modified Seurat Mitotic Sort (MoSMiS) code for the separation of mitotic and interphase cells from the G2/M population (Table 1 and Table 2).

Testing functional gene ontologies revealed that the most significantly enriched functional pathway in the “mitotic” genes was “microtubule, tubulin binding or other cytoskeletal protein binding”. Other terms enriched in this set involved the kinetochore and anaphase-promoting complex and ATPase and microtubule motor proteins (Table 3). We were, therefore, confident that this gene list represented a set of genes whose expression was higher in mitosis than in interphase and might make a suitable set of markers for identifying mitotic cells.

**Figure 4:**
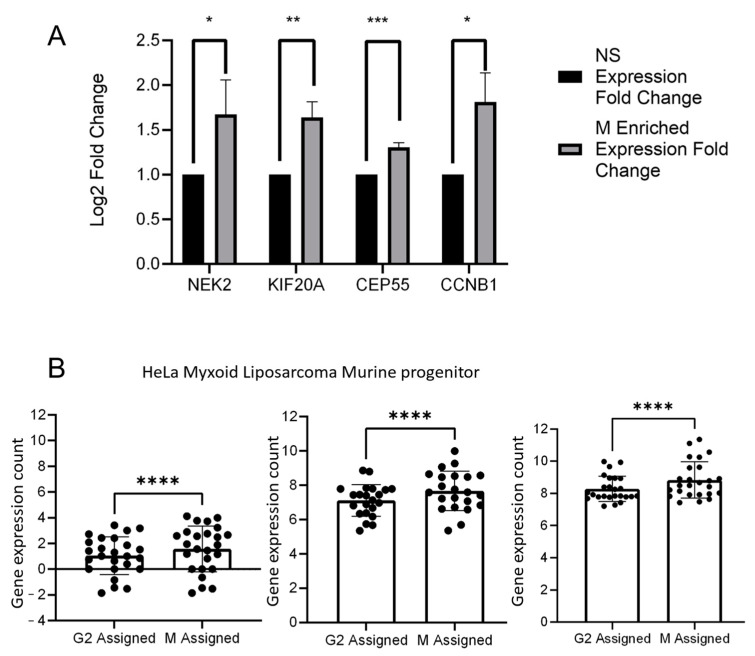
Validation of mitotic genes list. (**A**) HeLa cells were cultured for 24 h at 37 degrees Celsius and then separated by mitotic shake-off. qRTPCR was carried out on cDNA from RNA extracted from interphase (attached) or mitotic (shaken-off) HeLa cells. (**B**) Four-fold validation tests of gene exclusion list expression levels on log2 count matrix. Three online scRNAseq datasets were sorted into G2 and M phases of the cell cycle using MoSMiS with only 75% of the gene list. The expression of the remaining 25% of genes was then assessed in the two populations. *n* = 4 with a different 25% excluded from each run in order to test 100% of the genes. A paired *t*-test was performed compared to the corresponding control. * = *p* ≤ 0.05, ** = *p* ≤ 0.01, *** = *p* ≤ 0.001, **** = *p* ≤ 0.0001. Test datasets used: GSE129447-1, E-MTAB6142 and GSE81682.

To establish confidence in the gene lists generated by RNAseq analysis of mitotic vs. interphase cells, HeLa cells were separated into asynchronous and mitotically enriched populations using the mitotic shake-off method. This is an orthogonal method for separating mitotic and interphase cells and RTqPCR was carried out to assess the expression levels of the top four genes from our lists. If our gene lists derived from the RNAseq analysis of the flow cytometry sorted cells were accurate then we would expect to see elevated expression of the “mitotic” genes in the mitotically enriched fraction. All four of the genes selected showed elevated expression in the cells enriched for the mitotic population (Figure 4A), which gave confidence in the lists.

At this point we were able to use this gene set with MoSMiS to divide the G2/M population into G2 and M. To test this, we performed four-fold cross-validation tests using three external datasets: a murine haematopoietic stem cell/multipotent progenitor dataset from GSE81682 [19], a HeLa dataset from Hu et al. 2019 [17] (GSE129447) and a Myxoid Liposarcoma dataset (E-MTAB6142) [20]. In each case, the datasets were analysed with 25% of the interphase and mitotic-generated gene lists removed. This was performed four times removing a separate 25% of the gene each time. The expression levels of the removed genes in each of the four tests (interphase and mitotic tested separately) were then analysed for overall net expression in the mitotic and interphase populations which had been phase-assigned using the remaining 75% (Figure 4B). With all three datasets, and across all four folds, identifying M phase cells using 75% of the genes selected a group of cells with increased expression of the held out 25%.

**Figure 5:**
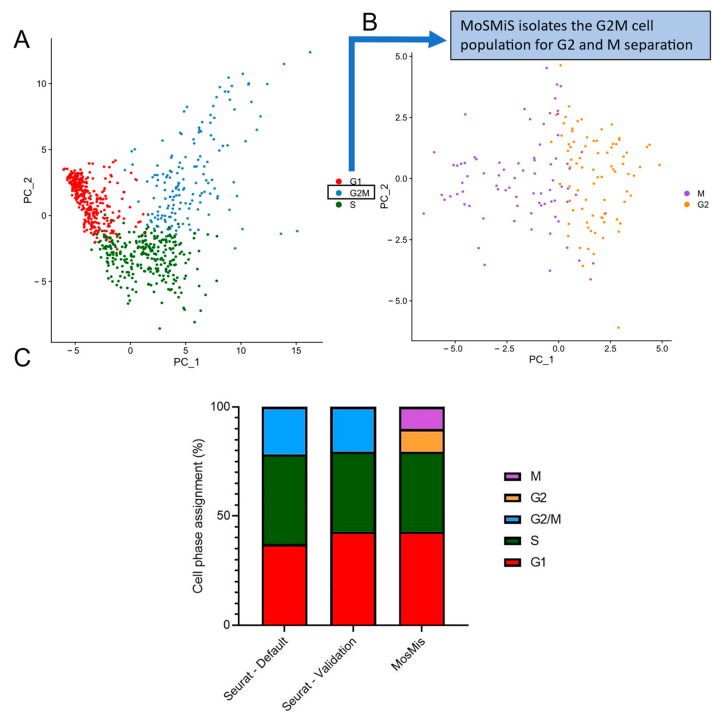
Comparative phase assignment from default Seurat Cell Cycle Sorting and Modified Seurat Mitotic Sort. Dataset GSE81682 (murine progenitor cells) was used as the test dataset as this was presented with the Seurat vignette. (**A**) GSE81682 was assigned to G1, S and G2 using the published unmodified Seurat code [14] using RC normalisation. Sorting is given in the distributed percentage of cell cycle phase assignments across the input data seen in both the plotted per cell PCA data and the total cell phase percentages. PCA shown is calculated from all input cells from GSE81682 using the Suerat G2/M and S separation gene of interest sets. (**B**) The same dataset was then further analysed using MoSMiS to add a further stage of Cell Cycle Scoring on the isolated G2/M cell population. PCA was calculated using the generated G2 and M-specific separation genes of interest set on the subsetted G2/M cells assigned in (**A**). (**C**) Shows the cell cycle assignment as dictated by the Seurat vignette. Tests were completed in R (https://www.r-project.org/ accessed 21 October 2022) using the Modified Seurat Mitotic Sort (MoSMiS) against the default Seurat phase assignment.

Following validation of our gene lists, we demonstrate phase assignment of an scRNAseq dataset GSE81682. The first step of the analysis follows the original Seurat procedure to separate G2/M from G1 and S phase cells, (Figure 5A) followed by a second division, this time of the G2/M assigned population into grouped G2 and mitotic populations (Figure 5B) using modified code. Principle component analysis of the G2/M cell identified by Seurat using our G2 and M markers shows separation along the first axis of variation of the cells we identify as G2 and M, adding confidence that they are in different cell cycle phases. The same dataset was used to demonstrate cell cycle sorting in the Seurat vignette [21], and we show their results in Figure 5C (Column 1). We first replicated their phase assignment, using the unaltered Seurat code as shown in Figure 5C (Column 2). Our data were within a few per cent of theirs for each phase of the cell cycle. Finally, Figure 5C (Column 3) shows that the same dataset with the G2/M population is now separated into distinct G2 and M populations.

## 3. Discussion

Using fluorescence-activated cell sorting of HeLa cells we were able to separate phospho-histone H3 positive cells from phospho-histone H3 negative cells. Histone H3 is phosphorylated on Serine 10 in mitosis, correlating directly with chromosome condensation [22,23]. The pH3 marker has been shown to be specific for mitotic cells with good reproducibility [24].

Mitosis is a fundamental period within the cell cycle whereby newly replicated DNA is accurately partitioned and segregated into identical daughter cells. Despite this, many aspects of this vital stage remain unknown. For example, whilst the DNA damage response is widely characterised in interphase cells, there is far less known about how cells respond to DNA breaks in mitosis [25]. Likewise, it has long been demonstrated that prolonged mitosis can lead to programmed cell death; however, exactly how this occurs is poorly understood [26,27]. However, p53 has been shown to be involved [27], indicating that there is a transcriptional aspect. Transcription in mitosis is also poorly understood as until recently it was assumed that transcription was largely repressed in mitosis [10,11,12]. Being able to study the transcriptional landscape of mitotic cells through bioinformatics is, therefore, incredibly beneficial to these fields.

MoSMiS could also provide new ways of answering other important questions about mitosis. For example, the study of whether various inherited mutations or epigenetic changes affect mitotic signalling or whether other environmental changes such as temperature and nutrient availability affect mitotic signalling. It would also be interesting to study mitosis in the presence of tumour-treating fields, an emerging treatment for cancer treatment which utilises alternating magnetic fields to disrupt mitosis [28].

MoSMiS can also have applications for other diseases and genetic conditions. Human autosomal trisomy 8, 13, 15, 16, 18, and 21 have all attributed causal links to mitotic separation errors [29,30,31] resulting from spindle assembly checkpoint (SAC) errors and even errors in cell division resulting from cytokinesis failures [31]. Isolating the mitotic cells from scRNAseq profiles from paired cell lines with and without engineered polysomies would be a good starting experiment to see how various trisomies impact mitotic division and then move onto scRNAseq datasets from patients with the trisomy compared to family members without.

Furthermore, with some slight modifications, we can see far-reaching applications of this tool. Segregation errors can have a major impact on the production of aberrant reproductive gamete cells contributing to birth defects [32]. Tweaks in the gene list could allow MoSMiS to separate meiotic cells in gamete progenitor cells for the study of meiotic signalling and gametogenesis. Thus, meiosis could be studied in reproductively challenged individuals to assess potential defects in gametogenesis. In summary, we have modified Seurat v4 to develop a tool for cell cycle phase assignment which is able to distinguish between G2 and M phase cells allowing for greater depths of analysis of single-cell RNAseq datasets going forward.

## 4. Materials and Methods

### 4.1. Cell Culture

HeLa cells (ATCC) were cultured in DMEM supplemented with 10% FBS at 37 °C + 5% CO_2_. Cells were passaged 1:10 every 3 to 4 days. Early passage HeLa cells (acquired from ATCC in the last 6 months) were used to ensure continuity with the GSE129447 dataset.

### 4.2. Fluorescence-Activated Cell Sorting

Cells were harvested using Trypsin EDTA (Lonza, Manchester, UK), washed in phosphate-buffered saline (PBS) and fixed for 30 min at −20 °C in 70% ethanol containing RNaseOUT™ Recombinant Ribonuclease Inhibitor solution (Invitrogen, #10777019, Waltham, MA, USA). The cells were then washed and rehydrated in PBS before incubation for 1 h on ice with Phospho-Histone H3 (Ser10) antibody (Sigma-Aldrich, St Louis, MO, USA) at 1:1000 in buffer 1 (PBS with 0.5% BSA and 0.25% Triton-X 100). The samples were washed in flow buffer 2 (500 mL PBS, 0.25% Triton-X 100) prior to incubation on ice for 30 min in the dark with secondary antibody pAb to Rabbit IgG (FITC) (Abcam, Cambridge, UK) 4:1000 in buffer 1. Following two washes in PBS, cells were incubated for 30 min in 5 mg/mL propidium iodide (Thermo-Fisher Scientific, Waltham, MA, USA) prior to cell sorting using FACSMelody (BD Biosciences, Allschwil, Switzerland). Cells were gated and sorted based on FITC staining levels (to sort mitotic vs. interphase cells) directly into the DNA/RNA lysis buffer (Zymo Research, Freiburg, Germany). In order to maintain RNA quality in the fixed cells, cells were kept on ice at all times and RNAseOUT was added to all buffers and solutions.

RNA was extracted using Zymo Research Quick-DNA/RNA Miniprep RNA extraction kit according to the manufacturer’s instructions. RNA quality was assessed using a total RNA Pico Agilent Bioanalyzer Chip and analysed using Agilent 2100 expert software to calculate RNA integrity numbers (RIN). Only RNA samples with RIN > 8 were used. RNA samples were stored at −80 °C and sent to Novogene (Cambridge, UK), where total RNA extract was purified to mRNA by poly(A) enrichment. Unstranded paired-end bulk RNA sequencing was performed on the Illumina NovaSeq PE150 to a depth of 40 million paired reads and a fragment length of 150 bp generating 12 G raw data per sample. A total of 3 biological replicates per condition were generated.

### 4.3. RNAseq Analysis

Quality control of unaligned read datasets was performed using FastQC through the Galaxy Europe GUI [33] (https://usegalaxy.eu/. Accessed 21 October 2022). Reads were mapped against the Human Dec 2013 (GRCh38/hg38) (hg38) reference genome using HISAT2 [34] (Please see https://daehwankimlab.github.io/hisat2/. Accessed 21 October 2022). Samtools (Please see https://www.htslib.org/. Accessed 21 October 2022) were used to check alignment quality. The number of reads per gene was calculated using htseq-count [35] (Please see https://htseq.readthedocs.io/en/release_0.11.1/count.html. Access 21 October 2022) against the reference genome. Count matrices were collated and annotated with Ensembl canonical gene symbols and RefSeq IDs. PCA analysis, normalisation and visualisation were all carried out in R.

Differential expression testing was conducted using DEseq2 [36] (Please see https://bioconductor.org/packages/release/bioc/html/DESeq2.html. Accessed 21 October 2022). Lowly expressed genes and outliers were removed by independent hypothesis filtering of data and Cook’s distance. We selected differentially expressed genes between the interphase and mitotic fractions where the adjusted Wald test *p*-value < 0.001 and ±0.58-fold change, representing 1.5 times more or less gene expression. The differentially expressed genes were used to create lists of mitotic (upregulated) and interphase (downregulated) cells based on positive or negative Log2FoldChange values, respectively. After selecting differentially expressed genes based on adjusted *p*-value and Log2FoldChange Cutoffs we derived 27 “Mitotic” related genes of interest and 18 “Interphase” related genes of interest.

### 4.4. Testing Cell Phase Assignment Tools

Existing cell phase assignment protocols (Seurat and Cyclone) were tested first using the single-cell RNA sequencing count matrix with which their systems were developed (E-MTAB-5522 in the case of Cyclone and GSE81682 in the case of Seurat), and then using [17] (GSE129447) using the GSM3713084 HeLa 1 p9 dataset.

### 4.5. Modified Seurat Mitotic Sort Procedure

The Modified Seurat Mitotic Sort uses the cell cycle state assignment approach implemented in Seurat [9] as outlined in the Cell Cycle Scoring and Regression Vignette. The original procedure sorts scRNAseq data in the cell cycle phase using gene lists which are relevant to each phase. Therefore, it was relatively straightforward to modify the code by adding a step after the G1, S and G2/M sorting steps which utilised our gene lists to sort the G2/M population into G2 and M. The basic Modified Seurat Mitotic Sort step-wise outline is as follows (with steps 1–4 being the original Seurat programme [21]] and 5–8 the extended steps to give our modified version: Modified Seurat Mitotic Sort).

1. The count matrix is normalised via a relative count system with an appropriate scale factor the using Seurat NormalizeData function.
2. Variable features are found based on the counts for the marker gene data—S and G2/M in this first instance, using the Seurat function FindVariableFeatures
3. Principle component analysis using the scaled and centred counts for the variable S and G2/M marker genes is carried out. This is visualised to verify separation.
4. G2/M, S and G1 phases are assigned, based on G2/M and S phase variability scores using the Seurat function CellCycleScoring
5. The G2/M pool identified in step 4 then had steps 2–3 repeated and the lists of Interphase and M phase genes identified from the gene lists above
6. The M phase and G2 phases are assigned to the cells assigned G2/M in the first pass using a modified CellCycleScoring function which assigns G2 or M to the G2/M population using the lists of marker genes identified above (see Extended Code 1).
7. G2 and M assignments are combined with the original G1 and S assignments to assign all cells to the G1, S, G2 or M phases.
8. Final phase assignments are then outputted in csv format. Following each CellCycleScoring step, assignments and the genes driving these assignments were examined using the DimPlot and RidgePlot Seurat functions, respectively.

### 4.6. K-Fold Testing

In order to give confidence to the gene lists and our modified cell sorting tool, four-fold cross-validation tests were completed using a range of datasets including a murine haematopoietic stem cell/multipotent progenitor dataset from GSE81682 [19], a HeLa dataset from Hu et al. 2019 (GSE129447) and a Myxoid Liposarcoma dataset from [20] (E-MTAB6142).

Datasets were transformed using rlog or vst transformation and sorted using the Modified Seurat Mitotic Sort procedure detailed above except that 25% of either the interphase or mitotic generated gene list of interest were removed first. This was performed 4 times to ensure full coverage, removing a different 25% of the gene population of the interphase and mitotic genes each time. The expression of the removed 25% genes in each of the 4 tests was then analysed for overall net expression in the populations which had been assigned to G2 or M by the remaining 75%.

### 4.7. RT-qPCR

Mitotic and interphase cells were separated by mitotic shake-off and RNA was extracted using the Zymo RNA extraction kit according to the manufacturer’s instructions. cDNA was generated using an RNA to cDNA kit (Thermo Fisher Scientific, Waltham, MA, USA). cDNA was mixed with the specified primers and SYBR green PCR master mix (Thermo Fisher Scientific) and 40 cycles of real-time PCR were carried out on a QuantStudio 7 Pro Real-Time PCR System (Thermo Fisher Scientific). We performed 3 biological replicates of each condition, and qPCR was carried out for each of these in 3 technical replicates. Data were analysed using the ddCT method [37] normalised to the geometric mean of two housekeeping genes: 18S and HPRT1. Statistical analysis was conducted by a paired *t*-test, and the mitotic enriched samples were compared to the corresponding control. Data were analysed via Prism.

### 4.8. Gene Function Ontologies

The gene lists generated by our RNAseq analysis of mitotic vs. interphase cells were analysed by GOrilla for gene ontology. The total gene list from the bulk RNAseq count matrix was used as the background gene list. The “interphase” and “mitotic” related genes of interest seen in Table 1 were used as the gene sets for function GO analysis. A *p*-value threshold of 10^−3^ was selected for testing.

### 4.9. Data and Code Availability

The data for testing were accessed from the gene expression omnibus. The HeLa cell dataset is available under accession code GSE129447 and the murine progenitor dataset is available under the code GSE81682.

The completed Modified Seurat Mitotic Sort (MosMis) code is available in Supplementary Materials.

## Figures and Tables

**Table 1:** Differential gene expression testing via Deseq2 (mitotic genes). Gene list post statistical significance and expression change filtering. Canon gene symbols are used unless unnamed/novel in which case the Ensembl number is provided. The gene list was generated post-filtration of regulated expression levels (log2FoldChange < 0.58) and statistically significant differential expression (padj < 0.001).

Upregulated Genes (Mitotic)	padj	log2FoldChange	Function
CENPE	0.000664	1.125147	Centromere Protein E
KNL1	0.000959	1.038927	Kinetochore Scaffold 1
PIMREG	0.000959	0.999074	PICALM Interacting Mitotic Regulator
PLK1	0.000966	1.403274	Polo Like Kinase 1
KIF14	0.000966	0.95761	Kinesin Family Member 14
TPX2	0.001844	1.33757	TPX2 Microtubule Nucleation Factor
KIF20A	0.002307	1.579072	Kinesin Family Member 20A
SAPCD2	0.003489	1.203906	Suppressor APC Domain Containing 2
KNSTRN	0.003702	1.225519	Kinetochore Astrin (SPAG5) Binding Protein
PRR11	0.003702	1.214335	Proline Rich 11
NUF2	0.003702	0.79701	NUF2 Component Of Kinetochore Complex
ASPM	0.004089	1.173306	Assembly Factor For Spindle Microtubules
CEP55	0.004128	1.546348	Centrosomal Protein 55
BUB1	0.004764	1.183972	BUB1 Mitotic Serine/Threonine Kinase
SGO2	0.005583	1.367488	Shugoshin 2
GAS2L3	0.005583	0.932372	Growth Arrest Specific 2 Like 3
NEK2	0.005939	1.737096	NIMA Related Kinase 2
HMMR	0.005939	1.470353	Hyaluronan Mediated Motility Receptor
DEPDC1	0.006015	1.184454	DEP Domain Containing 1
DLGAP5	0.007283	1.286611	DLG Associated Protein 5
ARL6IP1	0.007283	1.211374	ADP Ribosylation Factor Like GTPase 6 Interacting Protein 1
NUSAP1	0.007283	0.770366	Nucleolar and Spindle Associated Protein 1
CCNA2	0.007506	1.448108	Cyclin A2
VANGL1	0.008325	0.875813	VANGL Planar Cell Polarity Protein 1
CDC20	0.008993	1.462092	Cell Division Cycle 20
KIF4A	0.009799	1.454416	Kinesin Family Member 4A
KIF20B	0.01	1.224597	Kinesin Family Member 20B

**Table 2:** Differential gene expression testing via Deseq2 (interphase genes). Gene list post statistical significance and expression change filtering. Canonical gene symbols are used unless unnamed/novel in which case the Ensembl number is provided. The gene list was generated post-filtration of regulated expression levels (log2FoldChange < 0.58) and statistically significant differential expression (padj < 0.001).

Downregulated Genes (Interphase)	padj	log2FoldChange	Function
E2F1	2.84 × 10^−7^	−2.17369	E2F Transcription Factor 1
CCNE1	2.97 × 10^−5^	−2.2646	Cyclin E1
FBXL20	0.000966	−1.54022	F-box And Leucine-Rich Repeat Protein 20
DTL	0.000966	−1.53821	Denticleless E3 Ubiquitin Ligase Homolog
ENSG00000273759	0.001844	−2.59207	Uncategorised
RMI2	0.001844	−1.63698	Recq Mediated Genome Instability 2
ZMYND19	0.003489	−0.79722	Zinc Finger Mynd-Type Containing 19
MCM5	0.003702	−1.53998	Minichromosome Maintenance Component 5
ZNF367	0.003809	−1.44647	Zinc Finger Protein 367
FRAT1	0.004089	−1.81741	FRAT Regulator Of WNT Signalling Pathway 1
BRD2	0.005583	−1.7275	Bromodomain Containing 2
ENSG00000272106	0.007283	−2.14137	Uncategorised
PPP1R3C	0.007283	−1.51771	Protein Phosphatase 1 Regulatory Subunit 3C
ENSG00000275484	0.008993	−2.31626	Uncategorised
UNG	0.009006	−1.44849	Uracil DNA Glycosylase
IFI27L1	0.009278	−2.29293	Interferon Alpha Inducible Protein 27 Like 1
CDC6	0.009799	−0.95402	Cell Division Cycle 6

**Table 3:** Gene ontology function links for mitotic-related genes of interest. Microtubule, tubulin binding or other cytoskeletal protein binding all logically link a gene list related to mitotic function and expected roles in mitosis.

Mitotic Gene of Interest Grouped via GO Function			
GO Linked Ontology	*p*-Value	Ensembl	Gene Symbol
Anaphase-Promoting Complex Binding (GO Function)	6.06 × 10^−5^	ENSG00000117399. ENSG00000166851.	CDC20. PLK1.
ATP Binding (GO Function)	6.0 × 10^−4^	ENSG00000112984. ENSG00000138182. ENSG00000118193. ENSG00000090889. ENSG00000166851. ENSG00000169679. ENSG00000117650. ENSG00000138778.	KIF20A. KIF20B. KIF14. KIF4A. PLK1. BUB1. NEK2. CENPE.
Kinetochore (GO Component)	7.67 × 10^−12^	ENSG00000166851. ENSG00000169679. ENSG00000143228. ENSG00000138778. ENSG00000117650. ENSG00000163535. ENSG00000128944. ENSG00000137812.	PLK1. BUB1. NUF2. CENPE. NEK2. SGOL2. KNSTRN. CASC5.
Microtubule Motor Activity (GO Function)	3.73 × 10^−8^	ENSG00000138182. ENSG00000138778. ENSG00000112984. ENSG00000118193. ENSG00000090889.	KIF20B. CENPE. KIF20A. KIF14. KIFA.
Microtubule Binding (GO Function)	3.28 × 10^−14^	ENSG00000118193. ENSG00000112984. ENSG00000138182. ENSG00000090889. ENSG00000128944. ENSG00000137804. ENSG00000166851. ENSG00000088325. ENSG00000138778. ENSG00000126787. ENSG00000139354.	KIF14. KIF20A. KIF20B. KIF4A. KNSTRN. NUSAP1. PLK1. TPX2. CENPE. DLGAP5. GAS2L3.
Tubulin Binding (GO Function)	9.51 × 10^−13^	ENSG00000118193. ENSG00000112984. ENSG00000138182. ENSG00000090889. ENSG00000128944. ENSG00000137804. ENSG00000166851. ENSG00000088325. ENSG00000138778. ENSG00000126787. ENSG00000139354.	KIF14. KIF20A. KIF20B. KIF4A. KNSTRN. NUSAP1. PLK1. TPX2. CENPE. DLGAP5. GAS2L3.
Mitotic Spindle Pole (GO Component)	1.61 × 10^−5^	ENSG00000166851. ENSG00000138182. ENSG00000066279.	PLK1. KIF20B. ASPM.
Protein Binding (GO Function)	9.12 × 10^−4^	ENSG00000169679. ENSG00000129195. ENSG00000072571. ENSG00000173218. ENSG00000163535. ENSG00000128944. ENSG00000118193. ENSG00000138180. ENSG00000186193. ENSG00000166851. ENSG00000126787. ENSG00000137804. ENSG00000143228. ENSG00000145386. ENSG00000090889. ENSG00000137812. ENSG00000138182. ENSG00000170540. ENSG00000117650. ENSG00000066279. ENSG00000112984. ENSG00000088325. ENSG00000139354. ENSG00000117399. ENSG00000138778. ENSG00000024526.	BUB1. FAM64A. HMMR. VANGL1. SGOL2. KNSTRN. KIF14. CEP55. SAPCD2. PLK1. DLGAP5. NUSAP1. NUF2. CCNA2. KIF4A. CASC5. KIF20B. ARL6IP1. NEK2. ASPM. KIF20A. TPX2. GAS2L3. CDC20. CENPE. DEPDC1.

## Data Availability

Publicly available datasets were analysed in this study. Link/accession number available within the text.

## Supplementary Materials

The following supporting information can be downloaded at: https://www.mdpi.com/article/10.3390/ijms25094589/s1.
