# Supplementary material for: Modification of Seurat v4 for the Development of a Phase Assignment Tool Able to Distinguish between G2 and Mitotic Cells
Source: Int J Mol Sci. 2024 Apr 23;25(9):4589. doi: 10.3390/ijms25094589 (PMC11083997; doi:10.3390/ijms25094589)

MosMis

Steven Watson

26/10/2022

Input packages required

Note this version used Ensembl gene inputs needs conversion from canon gene names to Ensembl assignment numbers to function

```
library(ggplot2)
library(readr)
library(dplyr)
library(tidyr)
library(devtools)
library(SingleCellExperiment)
library(doParallel)
library(foreach)
library(Seurat)
library(plyr)
library(tibble)
```

Test scRNA seq data set and naming structure

Count matrix is input we demonstrate this the data set from murine hematopoietic progenitors in the appropriate format (Nestorowa et al., Blood 2016). Data set used can be found at https://www.ncbi.nlm.nih.gov/geo/query/acc.cgi?acc=GSE81682.

```
#Assign GSE or identifier - Will write with this prefix on output files
GSE <- "Nestorawa_"

#Read in count data
read_data <- read.csv("data/nestorawa_forcellcycle_expressionMatrix_Ensembl.c
sv", header = TRUE,
    as.is = TRUE , row.names = 1)

#Assigns column numbers for npcs used in RunPCA Functions
npcs_num <- ncol(read_data)
if (npcs_num  < 50) {
npcs_use <- npcs_num-1
} else {
npcs_use <- 50
}
```

Intial G1, S and G2/M Seurat cell phase assignment

We intial assign the input count matrix G1, S and G2/M phase assignment using the Seurat Cell Cycle Scoring function with relative count normalization. Seurat functions derived from https://satijalab.org/seurat/articles/cell_cycle_vignette.html.

Chosen input in this step uses the default seurat provided s and G2M genes to isolate a G2M only fraction using Ensembl Seurat Generic Input - converted from cc.genes.updated.20.

```
cell_marker_suerat <- read_csv("data/Seurat Genes Ensembl.csv")
seurat.s.genes <- cell_marker_suerat$seurat.s.genes
seurat.g2m.genes <- cell_marker_suerat$seurat.g2m.genes
seurat.s.genes <- na.omit(seurat.s.genes)
seurat.g2m.genes <- na.omit(seurat.g2m.genes)
```

A Seurat object is created from raw data

```
#Create a Seurat object from raw data
rnaseq_data_seuratgenes <- CreateSeuratObject(counts = read_data)

## Warning: Data is of class data.frame. Coercing to dgCMatrix.
```

Normalisation step of count matrix using Relative counts, input G2M and S feature counts for each cell are divided bt total counts and scaled by scale.factor. RC uses no log transformation, in further steps will log G2M fraction do not want to repeat log function.

```
rnaseq_data_var_seuratgenes <- NormalizeData(
  rnaseq_data_seuratgenes,
  assay = "RNA",
  normalization.method = "RC",
  scale.factor = 10000,
  margin = 1,
  verbose = TRUE
)

## Normalizing layer: counts
```

A mean variability plot is used to assign outliers in data set, the selection method vst was chosen. Vst fits the relationship of log(variance) and log(mean) using local polynomial regression (loess). The function then standardizes the feature valued using the mean and expected variance from that fitted relationship. The standardized values are used to calculate the feature variance.

```
rnaseq_data_var_seuratgenes <- FindVariableFeatures(rnaseq_data_var_seuratgen
es, selection.method = "vst")

## Finding variable features for layer counts
```

Features are centered and scaled in the dataset.

```
rnaseq_data_var_seuratgenes <- ScaleData(rnaseq_data_var_seuratgenes, feature
s = rownames(rnaseq_data_seuratgenes))
```

```
## Centering and scaling data matrix
```

Post scaling a PCA dimensionality reduction is run. Uses npcs assigned at chunk start and uses variable features of input count matrix with default seurat gene list of interest. PrintPCAParams can be run for more detail.

```
rnaseq_data_var_seuratgenes <- RunPCA(rnaseq_data_var_seuratgenes, features =
VariableFeatures(rnaseq_data_var_seuratgenes), npcs = (npcs_use), ndims.print
= 1:10, nfeatures.print = 10)

## PC_ 1
## Positive:  ENSG00000111348, ENSG00000089327, ENSG00000102879, ENSG00000162
511, ENSG00000067225, ENSG00000106565, ENSG00000145287, ENSG00000174804, ENSG
00000136167, ENSG00000174059
## Negative:  ENSG00000167741, ENSG00000164932, ENSG00000164010, ENSG00000111
843, ENSG00000107789, ENSG00000187010, ENSG00000080819, ENSG00000147885, ENSG
00000112077, ENSG00000256269
## PC_ 2
## Positive:  ENSG00000197694, ENSG00000005381, ENSG00000085491, ENSG00000140
471, ENSG00000138772, ENSG00000140749, ENSG00000011600, ENSG00000197561, ENSG
00000023330, ENSG00000170571
## Negative:  ENSG00000205639, ENSG00000130203, ENSG00000172794, ENSG00000179
348, ENSG00000169062, ENSG00000162366, ENSG00000205336, ENSG00000186766, ENSG
00000070190, ENSG00000154188
## PC_ 3
## Positive:  ENSG00000124216, ENSG00000130592, ENSG00000180758, ENSG00000159
579, ENSG00000168811, ENSG00000182568, ENSG00000164054, ENSG00000186265, ENSG
00000101544, ENSG00000107447
## Negative:  ENSG00000105374, ENSG00000130203, ENSG00000179348, ENSG00000149
516, ENSG00000182264, ENSG00000140287, ENSG00000205639, ENSG00000085491, ENSG
00000197561, ENSG00000173404
## PC_ 4
## Positive:  ENSG00000066739, ENSG00000071575, ENSG00000178982, ENSG00000161
011, ENSG00000169062, ENSG00000240583, ENSG00000166046, ENSG00000165702, ENSG
00000112212, ENSG00000163874
## Negative:  ENSG00000137804, ENSG00000175063, ENSG00000185156, ENSG00000131
747, ENSG00000141076, ENSG00000117724, ENSG00000170312, ENSG00000072571, ENSG
00000148773, ENSG00000162852
## PC_ 5
## Positive:  ENSG00000117400, ENSG00000146281, ENSG00000108924, ENSG00000149
564, ENSG00000221852, ENSG00000101000, ENSG00000168938, ENSG00000059377, ENSG
00000177606, ENSG00000150593
## Negative:  ENSG00000170540, ENSG00000129170, ENSG00000138764, ENSG00000159
625, ENSG00000175063, ENSG00000100385, ENSG00000197616, ENSG00000160767, ENSG
00000143013, ENSG00000137804
## PC_ 6
## Positive:  ENSG00000081189, ENSG00000177272, ENSG00000105583, ENSG00000186
766, ENSG00000172382, ENSG00000041353, ENSG00000115956, ENSG00000081026, ENSG
00000062822, ENSG00000177606
## Negative:  ENSG00000140470, ENSG00000140287, ENSG00000101000, ENSG00000149
```

```
516, ENSG00000033170, ENSG00000135404, ENSG00000108924, ENSG00000182264, ENSG
00000163751, ENSG00000197561
## PC_ 7
## Positive:  ENSG00000030419, ENSG00000033170, ENSG00000112977, ENSG00000139
193, ENSG00000174059, ENSG00000135503, ENSG00000176463, ENSG00000122223, ENSG
00000185885, ENSG00000111846
## Negative:  ENSG00000105583, ENSG00000172382, ENSG00000005961, ENSG00000081
026, ENSG00000136449, ENSG00000041353, ENSG00000177606, ENSG00000161911, ENSG
00000165985, ENSG00000150593
## PC_ 8
## Positive:  ENSG00000136449, ENSG00000110375, ENSG00000182578, ENSG00000112
799, ENSG00000152804, ENSG00000139990, ENSG00000126759, ENSG00000134352, ENSG
00000165985, ENSG00000189182
## Negative:  ENSG00000172543, ENSG00000115738, ENSG00000112081, ENSG00000184
402, ENSG00000186895, ENSG00000204103, ENSG00000081059, ENSG00000108602, ENSG
00000211751, ENSG00000282173
## PC_ 9
## Positive:  ENSG00000146281, ENSG00000129757, ENSG00000101000, ENSG00000136
449, ENSG00000100385, ENSG00000112799, ENSG00000165072, ENSG00000018408, ENSG
00000110375, ENSG00000150048
## Negative:  ENSG00000174059, ENSG00000112977, ENSG00000119535, ENSG00000174
944, ENSG00000159579, ENSG00000124226, ENSG00000147852, ENSG00000182568, ENSG
00000105851, ENSG00000070190
## PC_ 10
## Positive:  ENSG00000127481, ENSG00000136449, ENSG00000188404, ENSG00000110
375, ENSG00000167850, ENSG00000011600, ENSG00000170571, ENSG00000182578, ENSG
00000126759, ENSG00000112977
## Negative:  ENSG00000019582, ENSG00000160781, ENSG00000197561, ENSG00000143
153, ENSG00000149516, ENSG00000143434, ENSG00000186265, ENSG00000124145, ENSG
00000158517, ENSG00000126353
```

A heat map of the points of variance PC1 to PC10 are plotted

```
DimHeatmap(rnaseq_data_var_seuratgenes, dims = c(1:10))
```

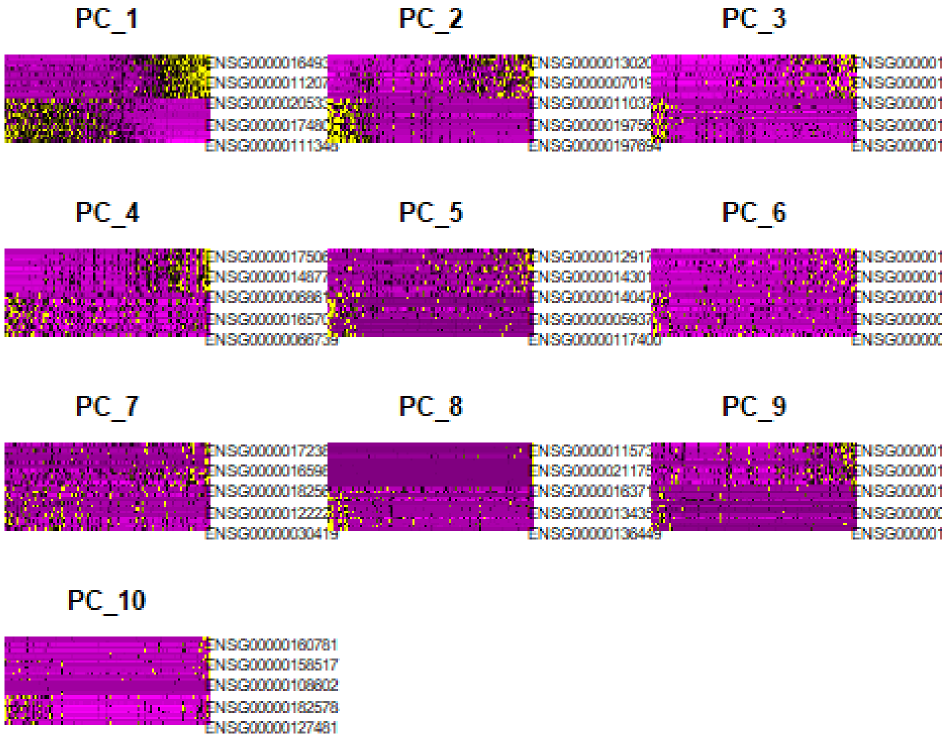

Cell Cycle Scoring uses default Seurat G2M and S genes of interest to assign each cell a score, based on its expression of G2/M and S phase markers. Cells expressing neither are likely not cycling and in G1 phase as the data set should be anticorrelated.

```
rnaseq_data_score_seuratgenes <- CellCycleScoring(rnaseq_data_var_seuratgenes
, s.features = seurat.s.genes, g2m.features = seurat.g2m.genes, set.ident = T
RUE)

## Warning: The following features are not present in the object: ENSG0000011
2312,
## not searching for symbol synonyms

## Warning: The following features are not present in the object: ENSG0000012
9195,
## ENSG00000189159, not searching for symbol synonyms
```

A PCA is generated from scored data without dimensionality reduction

```
DimPlot(object = rnaseq_data_score_seuratgenes, combine = FALSE, cols = c("re
d", "steelblue", "darkgreen"))

## [[1]]
```

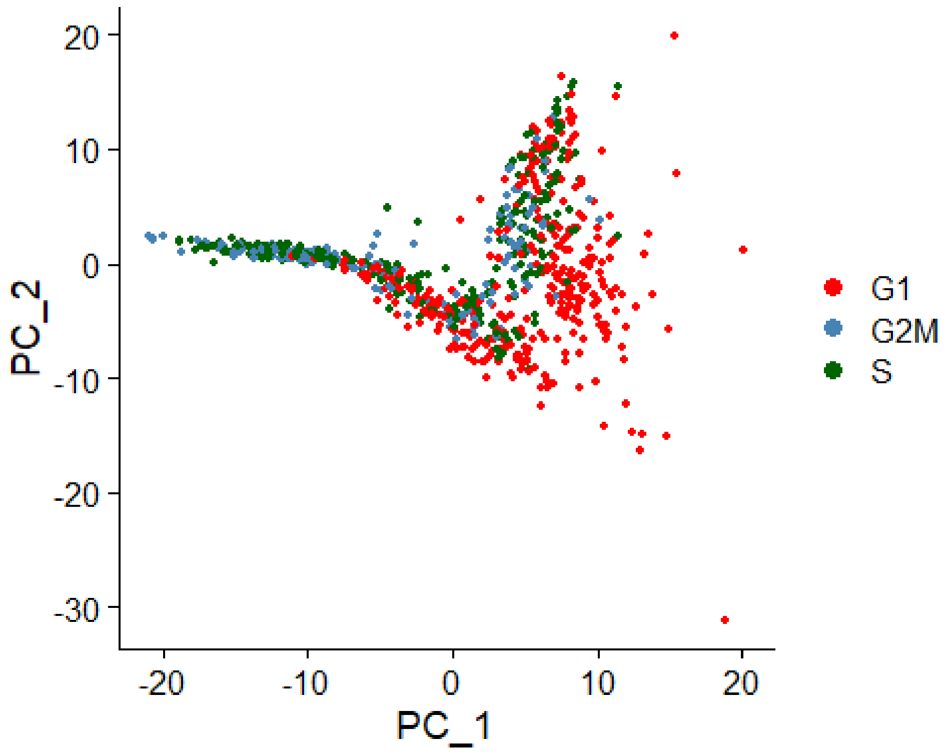

The top six results are printed to ensure proper output of phase assignment, S.Score and G2M.Score

```
head(rnaseq_data_score_seuratgenes[[]])

##          orig.ident nCount_RNA nFeature_RNA     S.Score G2M.Score Phase
## Prog_013       Prog    2029157         8366 -0.50854828 -1.381708    G1
## Prog_019       Prog    2408705         8194 -0.90542682  1.477430   G2M
## Prog_031       Prog    1033611         8349 -1.77053514 -1.295574    G1
## Prog_037       Prog    1068895         7955 -1.50509622  2.350027   G2M
## Prog_008       Prog    3174838         8596  2.37167494  0.304161     S
## Prog_014       Prog    1971269         8957 -0.07372224  1.050870   G2M
##          old.ident
## Prog_013      Prog
## Prog_019      Prog
## Prog_031      Prog
## Prog_037      Prog
## Prog_008      Prog
## Prog_014      Prog
```

Ridge plots of commonly seen genes are generated to ensure the proper differentiation of high S and high G2M scoring genes

```
RidgePlot(rnaseq_data_score_seuratgenes, features = c("ENSG00000117399", "ENS
G00000169679", "ENSG00000170312", "ENSG00000177302"), ncol = 2, cols = c("red
", "steelblue", "darkgreen"))
```

```
## Picking joint bandwidth of 0.5

## Picking joint bandwidth of 0.216

## Picking joint bandwidth of 0.59

## Picking joint bandwidth of 0.0478
```

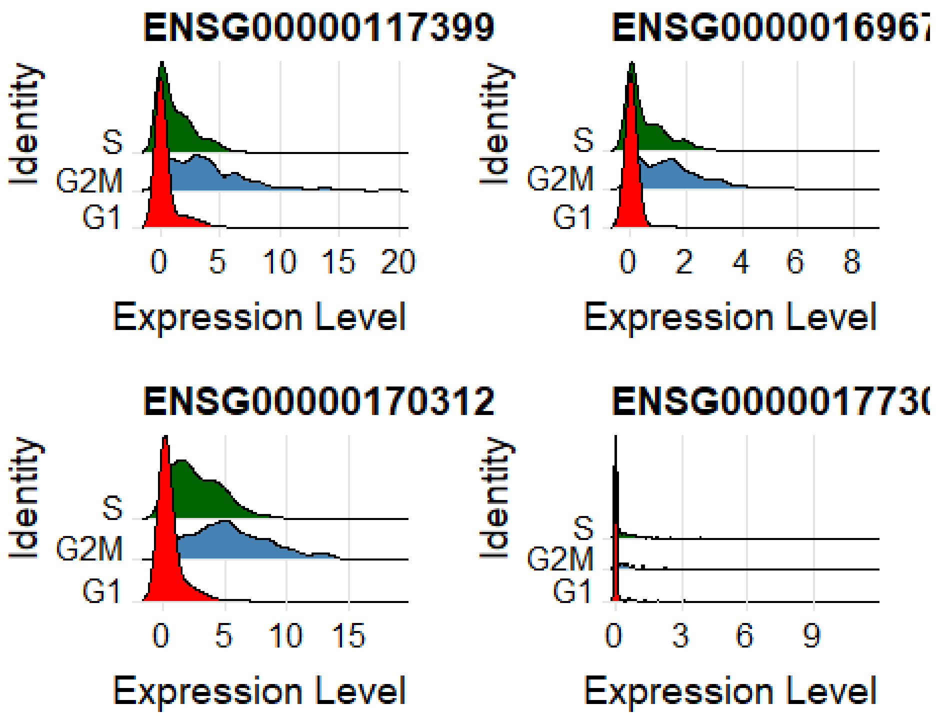

Generated and plotted PCA with proper grouping of phase scoring are produced

```
rnaseq_data_score_seuratgenes_pca <- RunPCA(rnaseq_data_score_seuratgenes, np
cs = (npcs_use), features = c(seurat.s.genes, seurat.g2m.genes))

## Warning in PrepDR5(object = object, features = features, layer = layer, :
The
## following features were not available: ENSG00000112312, ENSG00000129195,
## ENSG00000189159.

## Warning in irlba(A = t(x = object), nv = npcs, ...): You're computing too
large
## a percentage of total singular values, use a standard svd instead.

## PC_ 1
## Positive:  ENSG00000089685, ENSG00000111665, ENSG00000148773, ENSG00000170
312, ENSG00000131747, ENSG00000188229, ENSG00000134690, ENSG00000173207, ENSG
00000072571, ENSG00000175063
##     ENSG00000137804, ENSG00000013810, ENSG00000138160, ENSG00000113810, EN
SG00000178999, ENSG00000087586, ENSG00000115163, ENSG00000117399, ENSG0000008
```

```
8325, ENSG00000143228
##     ENSG00000167325, ENSG00000169679, ENSG00000142945, ENSG00000117724, EN
SG00000117650, ENSG00000126787, ENSG00000075218, ENSG00000157456, ENSG0000017
1848, ENSG00000176890
## Negative:  ENSG00000158402, ENSG00000174371, ENSG00000144354, ENSG00000076
248, ENSG00000169607, ENSG00000175216, ENSG00000138778, ENSG00000012963, ENSG
00000164104, ENSG00000095002
##     ENSG00000010292, ENSG00000118412, ENSG00000123975, ENSG00000134222, EN
SG00000129173, ENSG00000125630, ENSG00000123485, ENSG00000049541, ENSG0000018
4661, ENSG00000143476
##     ENSG00000102974, ENSG00000076003, ENSG00000119969, ENSG00000159259, EN
SG00000092853, ENSG00000104738, ENSG00000077514, ENSG00000092470, ENSG0000019
7299, ENSG00000139354
## PC_ 2
## Positive:  ENSG00000175063, ENSG00000137804, ENSG00000117724, ENSG00000142
945, ENSG00000088325, ENSG00000072571, ENSG00000137807, ENSG00000117399, ENSG
00000157456, ENSG00000138160
##     ENSG00000087586, ENSG00000134690, ENSG00000115163, ENSG00000092140, EN
SG00000138182, ENSG00000148773, ENSG00000117650, ENSG00000126787, ENSG0000016
9679, ENSG00000114346
##     ENSG00000131747, ENSG00000158402, ENSG00000136108, ENSG00000111665, EN
SG00000011426, ENSG00000102974, ENSG00000075218, ENSG00000113810, ENSG0000008
9685, ENSG00000143228
## Negative:  ENSG00000132646, ENSG00000276043, ENSG00000100297, ENSG00000076
003, ENSG00000075131, ENSG00000104738, ENSG00000166508, ENSG00000171848, ENSG
00000132780, ENSG00000163950
##     ENSG00000094804, ENSG00000131153, ENSG00000175305, ENSG00000119969, EN
SG00000076248, ENSG00000167325, ENSG00000051180, ENSG00000136982, ENSG0000019
8056, ENSG00000093009
##     ENSG00000168496, ENSG00000176890, ENSG00000159259, ENSG00000143476, EN
SG00000111247, ENSG00000156802, ENSG00000101868, ENSG00000049541, ENSG0000009
2470, ENSG00000077514
## PC_ 3
## Positive:  ENSG00000131747, ENSG00000101868, ENSG00000049541, ENSG00000178
999, ENSG00000176890, ENSG00000156802, ENSG00000120802, ENSG00000198056, ENSG
00000113810, ENSG00000080986
##     ENSG00000148773, ENSG00000094916, ENSG00000092470, ENSG00000102974, EN
SG00000011426, ENSG00000119969, ENSG00000138160, ENSG00000138182, ENSG0000019
7299, ENSG00000137804
##     ENSG00000170312, ENSG00000175216, ENSG00000051180, ENSG00000137807, EN
SG00000143476, ENSG00000123485, ENSG00000276043, ENSG00000114346, ENSG0000007
6003, ENSG00000143401
## Negative:  ENSG00000115163, ENSG00000157456, ENSG00000094804, ENSG00000175
305, ENSG00000168496, ENSG00000117399, ENSG00000171848, ENSG00000188229, ENSG
00000167325, ENSG00000171421
##     ENSG00000092853, ENSG00000139354, ENSG00000143815, ENSG00000112742, EN
SG00000100401, ENSG00000111665, ENSG00000132780, ENSG00000166508, ENSG0000010
4738, ENSG00000117650
##     ENSG00000075131, ENSG00000142945, ENSG00000089685, ENSG00000072571, EN
SG00000010292, ENSG00000087586, ENSG00000136108, ENSG00000134690, ENSG0000012
```

```
5630, ENSG00000163950
## PC_ 4
## Positive:  ENSG00000076248, ENSG00000143476, ENSG00000119969, ENSG00000049
541, ENSG00000136108, ENSG00000175216, ENSG00000076003, ENSG00000095002, ENSG
00000117399, ENSG00000162607
##      ENSG00000137807, ENSG00000117650, ENSG00000100297, ENSG00000104738, EN
SG00000010292, ENSG00000142945, ENSG00000157456, ENSG00000117724, ENSG0000016
6508, ENSG00000159259
##      ENSG00000137804, ENSG00000143815, ENSG00000175063, ENSG00000088325, EN
SG00000114346, ENSG00000126787, ENSG00000198056, ENSG00000276043, ENSG0000013
4222, ENSG00000138182
## Negative:  ENSG00000112742, ENSG00000176890, ENSG00000178999, ENSG00000089
685, ENSG00000111665, ENSG00000111247, ENSG00000171421, ENSG00000051180, ENSG
00000080986, ENSG00000125630
##      ENSG00000143228, ENSG00000171848, ENSG00000188229, ENSG00000148773, EN
SG00000132646, ENSG00000163950, ENSG00000167325, ENSG00000164104, ENSG0000013
1747, ENSG00000156802
##      ENSG00000102974, ENSG00000092853, ENSG00000197299, ENSG00000169607, EN
SG00000101868, ENSG00000158402, ENSG00000175305, ENSG00000094804, ENSG0000009
3009, ENSG00000184661
## PC_ 5
## Positive:  ENSG00000143815, ENSG00000095002, ENSG00000159259, ENSG00000168
496, ENSG00000077514, ENSG00000174371, ENSG00000167325, ENSG00000093009, ENSG
00000051180, ENSG00000076003
##      ENSG00000049541, ENSG00000137804, ENSG00000166508, ENSG00000013810, EN
SG00000137807, ENSG00000111247, ENSG00000011426, ENSG00000143228, ENSG0000017
8999, ENSG00000087586
##      ENSG00000163950, ENSG00000171848, ENSG00000158402, ENSG00000170312, EN
SG00000089685, ENSG00000114346, ENSG00000198056, ENSG00000188229, ENSG0000013
1153, ENSG00000176890
## Negative:  ENSG00000171421, ENSG00000162607, ENSG00000125630, ENSG00000143
401, ENSG00000197299, ENSG00000076248, ENSG00000092470, ENSG00000119969, ENSG
00000143476, ENSG00000123975
##      ENSG00000173207, ENSG00000132780, ENSG00000092853, ENSG00000115163, EN
SG00000120802, ENSG00000169679, ENSG00000100401, ENSG00000101868, ENSG0000016
4104, ENSG00000156802
##      ENSG00000118412, ENSG00000113810, ENSG00000112742, ENSG00000175216, EN
SG00000157456, ENSG00000144354, ENSG00000138182, ENSG00000117650, ENSG0000018
4661, ENSG00000075131
```

```r
DimPlot(rnaseq_data_score_seuratgenes_pca,combine = FALSE, cols = c("red", "s
teelblue", "darkgreen"))
```

```
## [[1]]
```

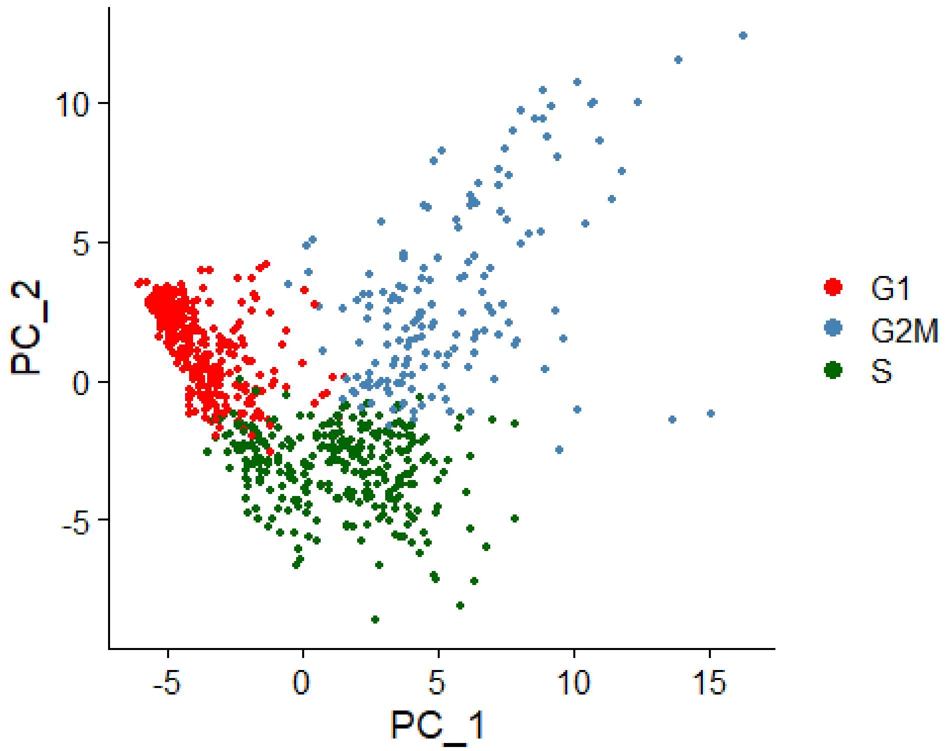

The phase percentages of G1, S and G2M are calculated and plotted

```
#Calculates and plots values of phase percentages of G1, S and G2M
phase_df <- rnaseq_data_score_seuratgenes_pca$Phase
phase_df <- count(phase_df)
names(phase_df)[1] <- 'Phase'
sumphase <- sum(phase_df$freq)
percentage_df <- phase_df$freq /sumphase *100
phase_df$Percentage <- percentage_df
phase_df <- phase_df %>%
  arrange(desc(Phase)) %>%
  mutate(prop = freq / sum(phase_df$freq) *100) %>%
  mutate(ypos = cumsum(prop)- 0.5*prop )
percent=(phase_df$Percentage)
percent=round(percent,digits=3)
phase_df$PercentageLabel <- paste0((percent), "%")

#Plots as a pie chart the assigned phase percentages in the overall intial co
unt matrix
ggplot(data = phase_df, aes(x = "", y = Percentage, fill = Phase)) +
  geom_bar(stat = "identity") +
  coord_polar("y")  + geom_text(aes(y = ypos, label = PercentageLabel), color
= "white", size=3)+ scale_fill_manual(values = c("G1"= "red", "G2M"="steelblu
e","S"= "darkgreen"))
```

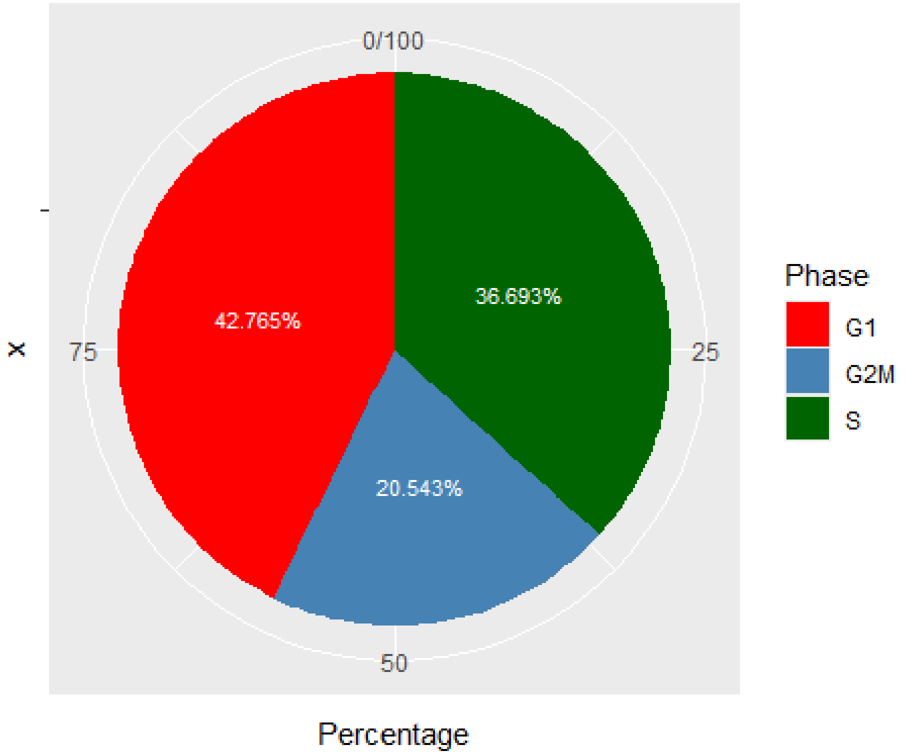

Tidying up data for G2 and M Seurat run We then isolate and subsets G2M, S and G1 into seperate variables so G2M can be processed further

```
rnaseq_seuratgenes_PCA_PhaseData <- FetchData(object = rnaseq_data_score_seur
atgenes, vars = c('orig.ident', 'nCount_RNA', 'nFeature_RNA', 'S.Score', 'G2M
.Score', 'Phase'))
rnaseq_seuratgenes_PCA_PhaseData<-tibble::rownames_to_column(rnaseq_seuratgen
es_PCA_PhaseData, "run")
rnaseq_seuratgenes_G2M <- filter(rnaseq_seuratgenes_PCA_PhaseData, Phase == "
G2M")
rnaseq_seuratgenes_S <-  filter(rnaseq_seuratgenes_PCA_PhaseData, Phase == "S
")
rnaseq_seuratgenes_G1 <-  filter(rnaseq_seuratgenes_PCA_PhaseData, Phase == "
G1")
```

The G2M scored data is tidied up which removes extra data so the G2M only fraction of input data can be ran through a modified Seurat phase assignment chunk to separate out a mitotic specific fraction

```
read_data_rotate <- data.frame(t(read_data[]))
read_data_rotate <- tibble::rownames_to_column(read_data_rotate)
names(read_data_rotate)[names(read_data_rotate) == "rowname"] <- "run"

joinfilter_g2m <- tibble::rownames_to_column(rnaseq_seuratgenes_G2M)

g2m_filtercells <- joinfilter_g2m$run
g2m_readdata <- filter(read_data_rotate, run %in% g2m_filtercells)
```

```
g2m_readdata <- data.frame(t(g2m_readdata[]))
names(g2m_readdata) <- as.matrix(g2m_readdata[1, ])
g2m_readdata <- g2m_readdata[-1, ]
```

Modification to CellCycleScoring for use in G2 and M phase assignment

Here we show the Seurat Cell Cycle Scoring function with the ability to assign G1 phase cells removed as this function would interfere with assigning phase to the G2/M subsetted cells into G2 and M specifically and ensure the proper labelling of said cells

```
CellCycleScoring_G1Disable <- function(
  object,
  s.features,
  g2m.features,
  ctrl = NULL,
  set.ident = FALSE,
  ...
) {
  name <- 'Cell.Cycle'
  features <- list('S.Score' = s.features, 'G2M.Score' = g2m.features)
  if (is.null(x = ctrl)) {
    ctrl <- min(vapply(X = features, FUN = length, FUN.VALUE = numeric(length
= 1)))
  }
  object.cc <- AddModuleScore(
    object = object,
    features = features,
    name = name,
    ctrl = ctrl,
    ...
  )
  cc.columns <- grep(pattern = name, x = colnames(x = object.cc[[]]), value =
TRUE)
  cc.scores <- object.cc[[cc.columns]]
  rm(object.cc)
  CheckGC()
  assignments <- apply(
    X = cc.scores,
    MARGIN = 1,
    FUN = function(scores, first = 'G2', second = 'M', null = 'G1') {
        if (length(which(x = scores == max(scores))) > 1) {
          return('Undecided')
        } else {
          return(c(first, second)[which(x = scores == max(scores))])
        }
      }
  )
  cc.scores <- merge(x = cc.scores, y = data.frame(assignments), by = 0)
  colnames(x = cc.scores) <- c('rownames', 'S.Score', 'G2M.Score', 'Phase')
  rownames(x = cc.scores) <- cc.scores$rownames
```

```
  cc.scores <- cc.scores[, c('S.Score', 'G2M.Score', 'Phase')]
  object[[colnames(x = cc.scores)]] <- cc.scores
  if (set.ident) {
    object[['old.ident']] <- Idents(object = object)
    Idents(object = object) <- 'Phase'
  }
  return(object)
}
```

G2 and M Seurat phase assignment

A second Seurat cell cycle phase assignment is run on log normalized cells using the generated Interphase and Mitotic gene list of interest, derived from the differentially expressed genes tested via Bulk RNA sequencing

```
#Reassigns column numbers for npcs used in RunPCA Functions for the G2M only
subsetted fraction
npcs_num <- ncol(g2m_readdata)

if (npcs_num  < 50) {
npcs_use_g2m <- npcs_num-1
} else {
npcs_use_g2m <- 50
}
```

Chosen input in this step uses generated gene list so the points of variance for phase assignment are Interphase (Regarded as G2 as they are derived from a G2M only fraction) and M Phase fraction

```
cell_marker_generated <- read_csv("RNAseq_sigGene_MvsI_Ensembl.csv")

## Rows: 27 Columns: 2
## ── Column specification ──────────────────────────────────────────────────
──────
## Delimiter: ","
## chr (2): Interphase_Padj, Mitotic_Padj
##
## ℹ Use `spec()` to retrieve the full column specification for this data.
## ℹ Specify the column types or set `show_col_types = FALSE` to quiet this m
essage.

g2_genes <- cell_marker_generated$Interphase_Padj
m_genes <- cell_marker_generated$Mitotic_Padj
g2_genes <- na.omit(g2_genes)
m_genes <- na.omit(m_genes)
```

A Seurat object is created from raw data

```
#Create a Seurat object from raw data
rnaseq_data_generatedgenes <- CreateSeuratObject(counts = g2m_readdata)
```

```
## Warning: Data is of class data.frame. Coercing to dgCMatrix.
```

Normalisation step of count matrix using Relative counts, input G2 and M feature counts for each cell are divided bt total counts and scaled by scale.factor. This is then natural-log transformed using log1p to now accurately scale the G2 and M variables respectively.

```
rnaseq_data_var_generatedgenes <- NormalizeData(
  rnaseq_data_generatedgenes,
  assay = "RNA",
  normalization.method = "LogNormalize",
  scale.factor = 10000,
  margin = 1,
  verbose = TRUE
)

## Normalizing layer: counts
```

A mean variability plot is used to assign outliers in data set, the selection method vst was chosen. Vst fits the relationship of log(variance) and log(mean) using local polynomial regression (loess). The function then standardizes the feature valued using the mean and expected variance from that fitted relationship. The standardized values are used to calculate the feature variance. In this step as stated G2 and M rather than S and G2M.

```
rnaseq_data_var_generatedgenes <- FindVariableFeatures(rnaseq_data_var_genera
tedgenes, selection.method = "vst")

## Finding variable features for layer counts
```

Features are centered and scaled in the dataset.

```
rnaseq_data_var_generatedgenes <- ScaleData(rnaseq_data_var_generatedgenes, f
eatures = rownames(rnaseq_data_generatedgenes))

## Centering and scaling data matrix
```

Post scaling a PCA dimensionality reduction is run. Uses npcs assigned at chunk start and uses variable features of input count matrix with default seurat gene list of interest. PrintPCAParams can be run for more detail.

```
rnaseq_data_var_generatedgenes <- RunPCA(rnaseq_data_var_generatedgenes, feat
ures = VariableFeatures(rnaseq_data_var_generatedgenes), npcs = (npcs_use_g2m
), ndims.print = 1:10, nfeatures.print = 10)

## PC_ 1
## Positive:  ENSG00000174804, ENSG00000142669, ENSG00000089327, ENSG00000145
287, ENSG00000153551, ENSG00000111348, ENSG00000130429, ENSG00000102879, ENSG
00000204264, ENSG00000162511
## Negative:  ENSG00000167741, ENSG00000164932, ENSG00000164010, ENSG00000107
789, ENSG00000187010, ENSG00000130783, ENSG00000240583, ENSG00000147885, ENSG
00000080819, ENSG00000112077
## PC_ 2
## Positive:  ENSG00000124216, ENSG00000112799, ENSG00000101544, ENSG00000110
```

```
375, ENSG00000140471, ENSG00000124217, ENSG00000107447, ENSG00000167460, ENSG
00000182568, ENSG00000158517
## Negative:  ENSG00000130203, ENSG00000179348, ENSG00000011295, ENSG00000186
766, ENSG00000005961, ENSG00000132622, ENSG00000165646, ENSG00000183785, ENSG
00000010278, ENSG00000172794
## PC_ 3
## Positive:  ENSG00000168685, ENSG00000113312, ENSG00000143344, ENSG00000185
437, ENSG00000163625, ENSG00000182866, ENSG00000170819, ENSG00000115607, ENSG
00000172995, ENSG00000134531
## Negative:  ENSG00000197561, ENSG00000085491, ENSG00000140749, ENSG00000257
017, ENSG00000197694, ENSG00000160883, ENSG00000138772, ENSG00000005381, ENSG
00000129988, ENSG00000149516
## PC_ 4
## Positive:  ENSG00000243063, ENSG00000169684, ENSG00000100147, ENSG00000172
426, ENSG00000236637, ENSG00000168878, ENSG00000170819, ENSG00000126106, ENSG
00000172995, ENSG00000106100
## Negative:  ENSG00000134061, ENSG00000141076, ENSG00000108861, ENSG00000091
157, ENSG00000183615, ENSG00000172382, ENSG00000112576, ENSG00000138303, ENSG
00000166831, ENSG00000187862
## PC_ 5
## Positive:  ENSG00000006638, ENSG00000164442, ENSG00000197943, ENSG00000135
253, ENSG00000154096, ENSG00000173928, ENSG00000183615, ENSG00000137101, ENSG
00000108861, ENSG00000149564
## Negative:  ENSG00000107036, ENSG00000262406, ENSG00000198771, ENSG00000171
757, ENSG00000142619, ENSG00000148814, ENSG00000196141, ENSG00000268223, ENSG
00000131094, ENSG00000005187
## PC_ 6
## Positive:  ENSG00000137845, ENSG00000117399, ENSG00000224189, ENSG00000163
874, ENSG00000111335, ENSG00000094975, ENSG00000185507, ENSG00000079337, ENSG
00000149534, ENSG00000157514
## Negative:  ENSG00000146281, ENSG00000163737, ENSG00000166831, ENSG00000172
382, ENSG00000120457, ENSG00000164220, ENSG00000187862, ENSG00000134061, ENSG
00000183615, ENSG00000108861
## PC_ 7
## Positive:  ENSG00000102760, ENSG00000125730, ENSG00000236279, ENSG00000063
660, ENSG00000124191, ENSG00000114487, ENSG00000149516, ENSG00000243414, ENSG
00000106066, ENSG00000187796
## Negative:  ENSG00000273749, ENSG00000215612, ENSG00000002549, ENSG00000106
077, ENSG00000076928, ENSG00000090339, ENSG00000134762, ENSG00000138378, ENSG
00000110375, ENSG00000143614
## PC_ 8
## Positive:  ENSG00000113312, ENSG00000179673, ENSG00000182957, ENSG00000228
741, ENSG00000273167, ENSG00000185189, ENSG00000148814, ENSG00000137845, ENSG
00000198053, ENSG00000112378
## Negative:  ENSG00000256590, ENSG00000119431, ENSG00000072840, ENSG00000170
476, ENSG00000175063, ENSG00000187416, ENSG00000167286, ENSG00000050730, ENSG
00000048462, ENSG00000079616
## PC_ 9
## Positive:  ENSG00000138161, ENSG00000167900, ENSG00000001497, ENSG00000067
836, ENSG00000219607, ENSG00000092964, ENSG00000085999, ENSG00000125910, ENSG
```

```
00000161960, ENSG00000166710
## Negative:  ENSG00000124191, ENSG00000125730, ENSG00000144677, ENSG00000106
066, ENSG00000104154, ENSG00000128944, ENSG00000110060, ENSG00000103569, ENSG
00000063660, ENSG00000083750
## PC_ 10
## Positive:  ENSG00000147168, ENSG00000147852, ENSG00000176571, ENSG00000104
341, ENSG00000123243, ENSG00000067191, ENSG00000176463, ENSG00000107281, ENSG
00000179097, ENSG00000181523
## Negative:  ENSG00000124571, ENSG00000048462, ENSG00000256590, ENSG00000119
431, ENSG00000170476, ENSG00000072840, ENSG00000211896, ENSG00000187416, ENSG
00000240041, ENSG00000130775
```

A heat map of the points of variance PC1 to PC10 are plotted

```
DimHeatmap(rnaseq_data_var_generatedgenes, dims = c(1:10))
```

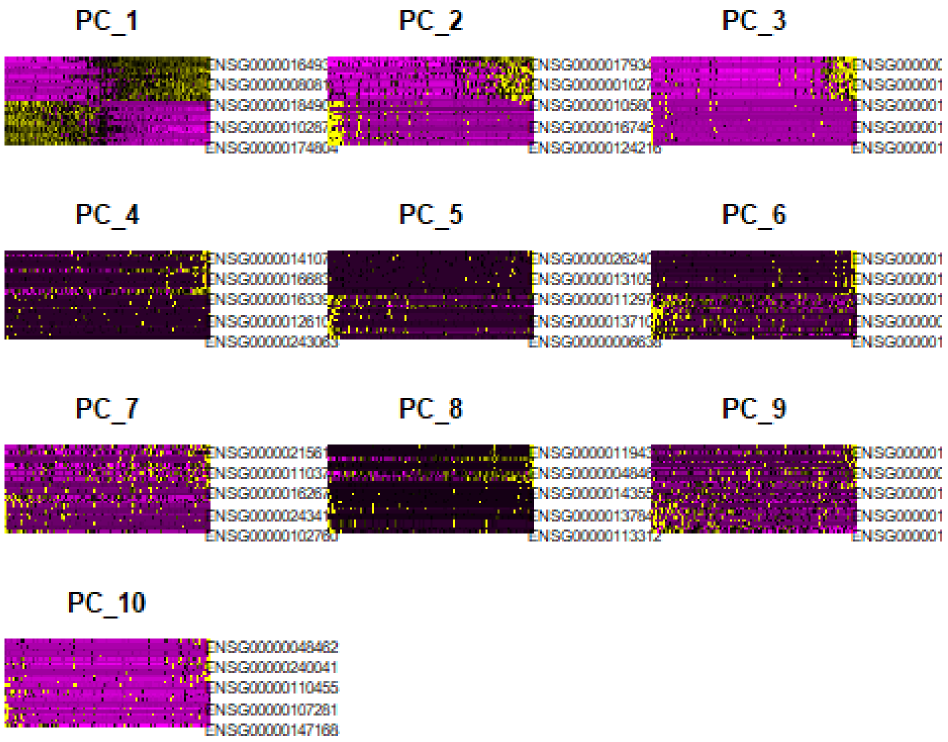

Cell Cycle Scoring uses CellCycleScoring_G1Disable to correctly assign cells based on its expression of G2 (Interphase) and M phase markers, G1 calling is not required in this step.

```
rnaseq_data_score_generatedgenes <- CellCycleScoring_G1Disable(rnaseq_data_va
r_generatedgenes, s.features = g2_genes, g2m.features = m_genes, set.ident =
TRUE)

## Warning: The following features are not present in the object: ENSG0000027
3759,
## ENSG00000165244, ENSG00000272106, ENSG00000275484, ENSG00000165948,
## ENSG00000207547, not searching for symbol synonyms
```

```
## Warning: The following features are not present in the object: ENSG0000012
9195,
## ENSG00000163535, ENSG00000024526, ENSG00000090889, not searching for symbo
l
## synonyms
```

A PCA is generated from scored data without dimensionality reduction

```
DimPlot(object = rnaseq_data_score_generatedgenes, combine = FALSE, cols = c(
"#E3E857", "#6a329f", "#8fce00"))

## [[1]]
```

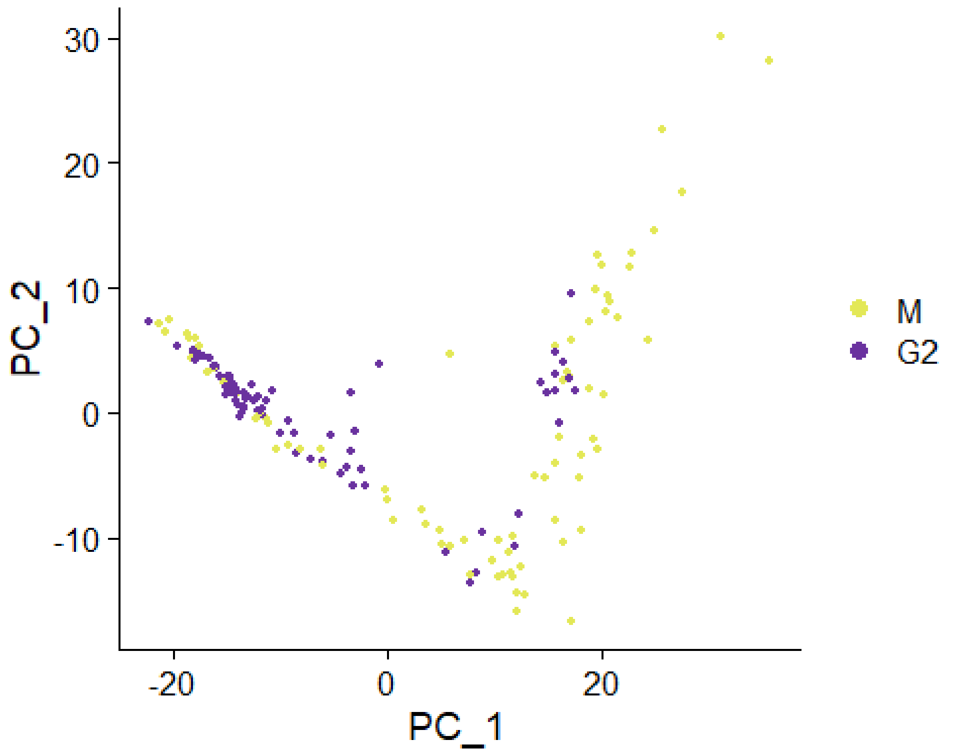

The top six results are printed to ensure proper output of phase assignment, S.Score (Modified to investigate G2 genes of interest) and G2M.Score (Modified to investigate M genes of interest)

```
head(rnaseq_data_score_generatedgenes[[]])

##          orig.ident nCount_RNA nFeature_RNA     S.Score   G2M.Score Phase
## Prog_019       Prog    2408705         8194 -0.17581863  0.17891918     M
## Prog_037       Prog    1068895         7955 -0.38620260  0.35113848     M
## Prog_014       Prog    1971269         8957  0.02925160 -0.07011165    G2
## Prog_032       Prog    1260663         8301  0.08545737 -0.16467122    G2
## Prog_038       Prog    1754706         8132  0.06324387 -0.29006825    G2
## Prog_009       Prog    3551293         8694  0.09625127 -0.34645865    G2
##          old.ident
## Prog_019      Prog
```

```
## Prog_037      Prog
## Prog_014      Prog
## Prog_032      Prog
## Prog_038      Prog
## Prog_009      Prog
```

Generated and plotted PCA with proper grouping of phase scoring are produced

```
rnaseq_data_score_generatedgenes_pca <- RunPCA(rnaseq_data_score_generatedgen
es, npcs = (npcs_use), features = c(g2_genes, m_genes))

## Warning in PrepDR5(object = object, features = features, layer = layer, :
The
## following features were not available: ENSG00000165244, ENSG00000272106,
## ENSG00000275484, ENSG00000165948, ENSG00000207547, ENSG00000129195,
## ENSG00000163535, ENSG00000024526, ENSG00000090889.

## Warning in irlba(A = t(x = object), nv = npcs, ...): You're computing too
large
## a percentage of total singular values, use a standard svd instead.

## Warning in irlba(A = t(x = object), nv = npcs, ...): did not converge--res
ults
## might be invalid!; try increasing work or maxit

## Warning: Requested number is larger than the number of available items (35
).
## Setting to 35.

## Warning: Requested number is larger than the number of available items (35
).
## Setting to 35.

## Warning: Requested number is larger than the number of available items (35
).
## Setting to 35.

## Warning: Requested number is larger than the number of available items (35
).
## Setting to 35.

## Warning: Requested number is larger than the number of available items (35
).
## Setting to 35.

## PC_ 1
## Positive:  ENSG00000105173, ENSG00000165879, ENSG00000094804, ENSG00000076
248, ENSG00000101412, ENSG00000165724, ENSG00000100297, ENSG00000204256, ENSG
00000139354, ENSG00000143476
##     ENSG00000175643, ENSG00000173218, ENSG00000119938, ENSG00000138778, EN
SG00000118193, ENSG00000108306, ENSG00000145386, ENSG00000137812
```

```
## Negative:  ENSG00000170540, ENSG00000137804, ENSG00000128944, ENSG00000117
399, ENSG00000088325, ENSG00000072571, ENSG00000138180, ENSG00000066279, ENSG
00000186193, ENSG00000138182
##     ENSG00000126787, ENSG00000068489, ENSG00000117650, ENSG00000166851, EN
SG00000112984, ENSG00000143228, ENSG00000169679
## PC_ 2
## Positive:  ENSG00000105173, ENSG00000166851, ENSG00000094804, ENSG00000145
386, ENSG00000100297, ENSG00000072571, ENSG00000117399, ENSG00000173218, ENSG
00000117650, ENSG00000139354
##     ENSG00000118193, ENSG00000165879, ENSG00000066279, ENSG00000112984, EN
SG00000137812, ENSG00000076248, ENSG00000165724, ENSG00000126787
## Negative:  ENSG00000138180, ENSG00000175643, ENSG00000138778, ENSG00000108
306, ENSG00000068489, ENSG00000128944, ENSG00000138182, ENSG00000101412, ENSG
00000119938, ENSG00000204256
##     ENSG00000143476, ENSG00000143228, ENSG00000137804, ENSG00000170540, EN
SG00000088325, ENSG00000169679, ENSG00000186193
## PC_ 3
## Positive:  ENSG00000137812, ENSG00000118193, ENSG00000108306, ENSG00000119
938, ENSG00000139354, ENSG00000076248, ENSG00000165879, ENSG00000088325, ENSG
00000186193, ENSG00000068489
##     ENSG00000138182, ENSG00000066279, ENSG00000166851, ENSG00000169679, EN
SG00000138778, ENSG00000094804, ENSG00000105173, ENSG00000128944
## Negative:  ENSG00000143476, ENSG00000175643, ENSG00000100297, ENSG00000117
650, ENSG00000101412, ENSG00000138180, ENSG00000204256, ENSG00000170540, ENSG
00000126787, ENSG00000112984
##     ENSG00000143228, ENSG00000137804, ENSG00000173218, ENSG00000145386, EN
SG00000117399, ENSG00000072571, ENSG00000165724
## PC_ 4
## Positive:  ENSG00000173218, ENSG00000066279, ENSG00000119938, ENSG00000175
643, ENSG00000138778, ENSG00000143476, ENSG00000169679, ENSG00000094804, ENSG
00000137812, ENSG00000137804
##     ENSG00000170540, ENSG00000186193, ENSG00000101412, ENSG00000072571, EN
SG00000108306, ENSG00000138182, ENSG00000068489, ENSG00000105173
## Negative:  ENSG00000145386, ENSG00000117399, ENSG00000138180, ENSG00000126
787, ENSG00000128944, ENSG00000112984, ENSG00000076248, ENSG00000165879, ENSG
00000139354, ENSG00000088325
##     ENSG00000143228, ENSG00000117650, ENSG00000204256, ENSG00000118193, EN
SG00000100297, ENSG00000165724, ENSG00000166851
## PC_ 5
## Positive:  ENSG00000143228, ENSG00000068489, ENSG00000204256, ENSG00000139
354, ENSG00000175643, ENSG00000137812, ENSG00000118193, ENSG00000166851, ENSG
00000137804, ENSG00000100297
##     ENSG00000126787, ENSG00000105173, ENSG00000138180, ENSG00000170540, EN
SG00000143476, ENSG00000138778, ENSG00000145386, ENSG00000066279
## Negative:  ENSG00000101412, ENSG00000186193, ENSG00000128944, ENSG00000173
218, ENSG00000117650, ENSG00000112984, ENSG00000169679, ENSG00000076248, ENSG
00000094804, ENSG00000117399
##     ENSG00000108306, ENSG00000072571, ENSG00000138182, ENSG00000165879, EN
SG00000165724, ENSG00000119938, ENSG00000088325
```

```
## Warning: Number of dimensions changing from 50 to 34
```

```
DimPlot(object = rnaseq_data_score_generatedgenes_pca, combine = FALSE, cols
= c("#984DD3", "#E88A13", "#8fce00"))
```

```
## [[1]]
```

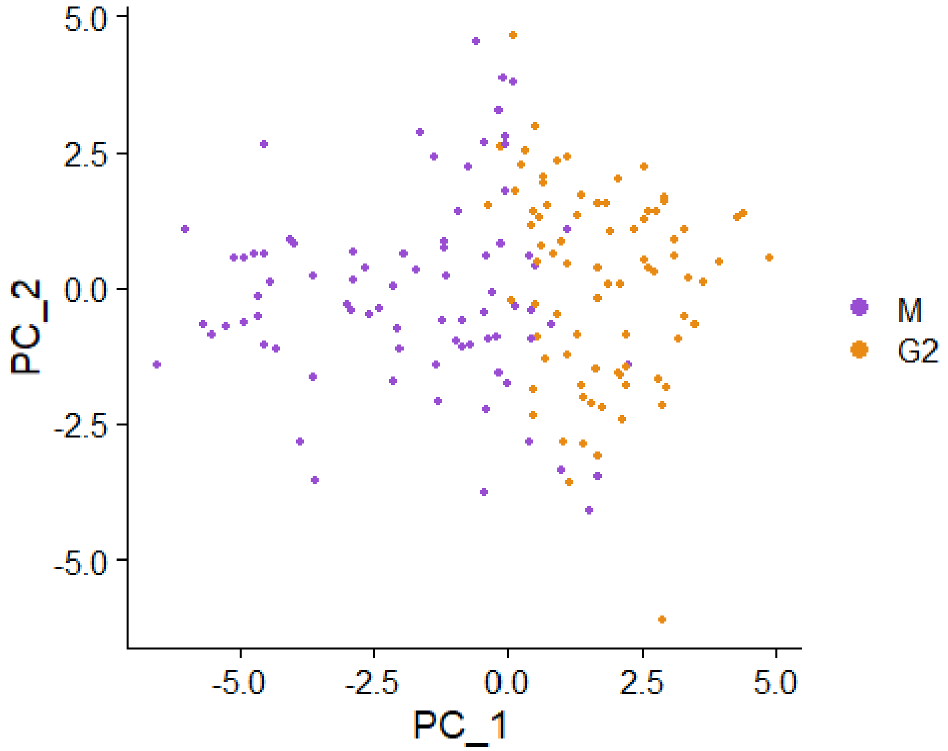

Plots commonly seen genes to ensure the proper expression of high G2 and high M scoring genes is present

```
#Plots commonly seen genes to ensure the proper differentiation of high G2 (r
epresenting the G2 cells) and high G2M scoring genes(representing the M cells
)
RidgePlot(rnaseq_data_score_generatedgenes, features = c("ENSG00000117399", "
ENSG00000169679", "ENSG00000170312", "ENSG00000177302"), ncol = 2, cols = c("
#984DD3", "#E88A13", "#8fce00"))
```

```
## Picking joint bandwidth of 0.228

## Picking joint bandwidth of 0.19

## Picking joint bandwidth of 0.193

## Picking joint bandwidth of 0.0714
```

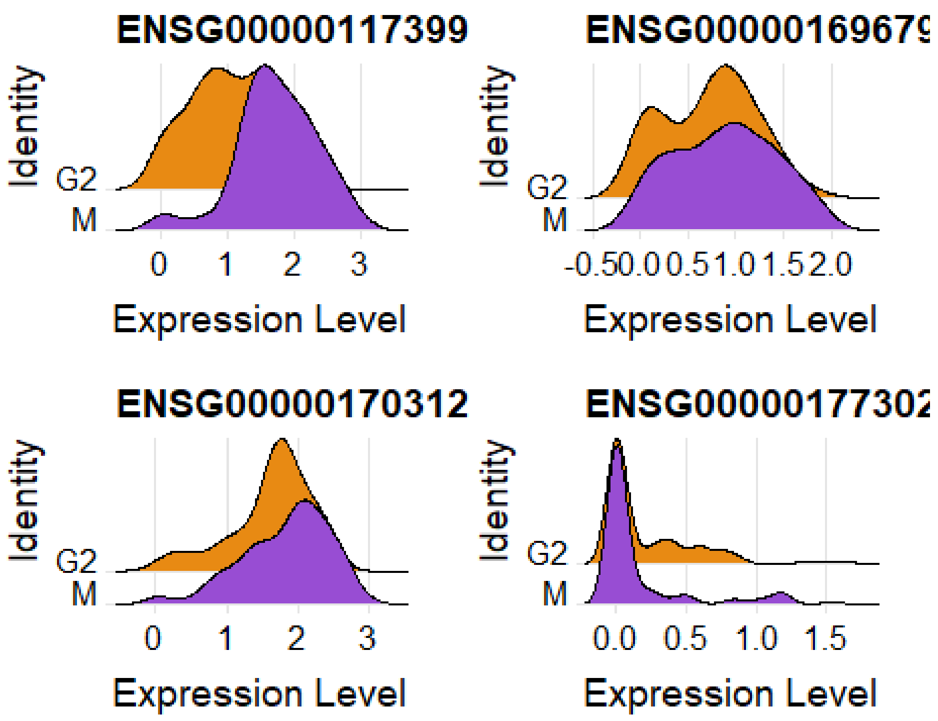

The phase percentages of G2 and M are calculated and plotted

```
phase_geninput <- rnaseq_data_score_generatedgenes$Phase
phase_geninput <- count(phase_geninput)
names(phase_geninput)[1] <- 'Phase'
sumphase <- sum(phase_geninput$freq)
percentage_df <- phase_geninput$freq /sumphase *100
phase_geninput$Percentage <- percentage_df
phase_geninput <- phase_geninput %>%
  arrange(desc(Phase)) %>%
  mutate(prop = freq / sum(phase_geninput$freq) *100) %>%
  mutate(ypos = cumsum(prop)- 0.5*prop )
percent=(phase_geninput$Percentage)
percent=round(percent,digits=3)
phase_geninput$PercentageLabel <- paste0((percent), "%")

#Plots as a pie chart the assigned phase percentages in the overall intial co
unt matrix
ggplot(data = phase_geninput, aes(x = "", y = Percentage, fill = Phase)) +
  geom_bar(stat = "identity") +
  coord_polar("y")  + geom_text(aes(y = ypos, label = PercentageLabel), color
= "white", size=3) + scale_fill_manual(values = c("M"= "#984DD3", "G2"="#E88A
13"))
```

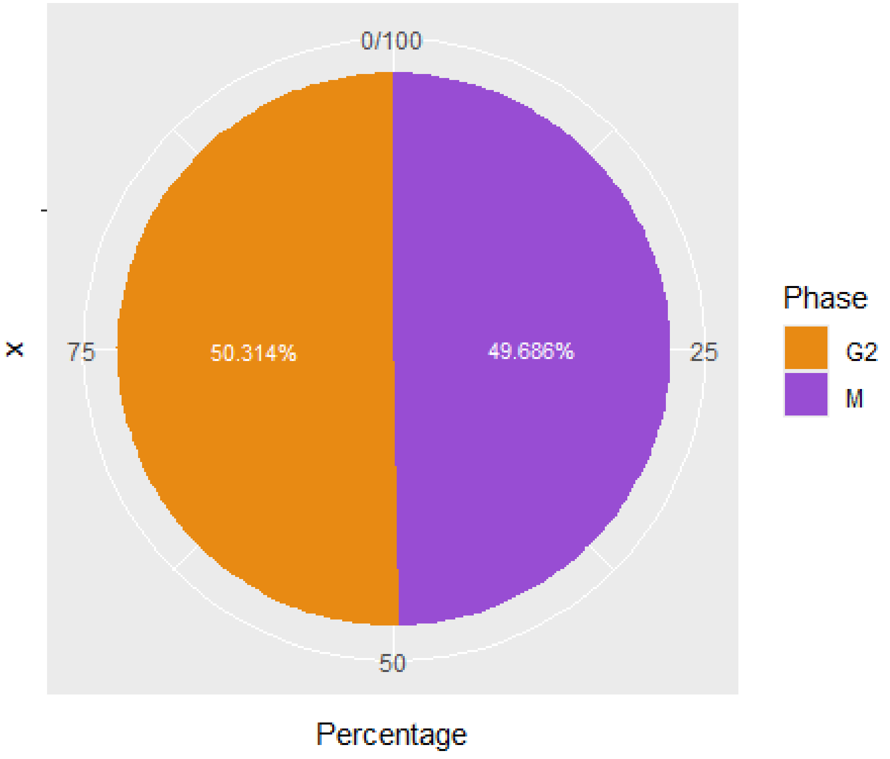

To ensure that there is no error in naming we added this function to ensure that the G2 and M assigned cells in the Seurat G2 and M phase assignment would always be correct

```
g2m_cellcyclescored <- FetchData(object = rnaseq_data_score_generatedgenes, vars = c('orig.ident', 'nCount_RNA', 'nFeature_RNA', 'S.Score', 'G2M.Score', 'Phase'))
g2m_cellcyclescored$Phase <- revalue(g2m_cellcyclescored$Phase, c("G2M"="M"))

## The following `from` values were not present in `x`: G2M

g2m_cellcyclescored$Phase <- revalue(g2m_cellcyclescored$Phase, c("S"="G2"))

## The following `from` values were not present in `x`: S
```

The G2 and M scored cells are the rejoined with the intial count matrix to generated a new matrix containing only G2 and M scored genes post phase assignment from the G2 and M Seurat sort

```
#The now renamed G2 and M scored cells are the rejoined with the intial count matrix to generated a new matrix containing G2 and M scored genes from the SW generated Seurat sort in the previous chunk
g2_m_finalsplit <- g2m_cellcyclescored %>% rownames_to_column("run")
g2_m_phase_Counts <- left_join(g2_m_finalsplit, read_data_rotate, by = "run")
g2_m_phase_Counts <- subset(g2_m_phase_Counts, select = -c(orig.ident, nCount_RNA, nFeature_RNA, S.Score, G2M.Score))
g2_m_phase_Counts <- data.frame(t(g2_m_phase_Counts[]))
names(g2_m_phase_Counts) <- as.matrix(g2_m_phase_Counts[1, ])
g2_m_phase_Counts <- g2_m_phase_Counts[-1, ]
```

If required this write out function can be enabled to generate this G2 and M specific count matrix

```
write.csv(g2_m_phase_Counts, file = (paste((GSE),"G2_and_M_Phases_Assigned.cs
v")))
```

Collation of phase assignment and graphing

Here we combine all phase assigned variables together to get a count matrix with G1, G2, S and M assigned cells and write out a complete count matrix with phase assignment attached

```
#Combined all phase assigned variables together to get a count matrix with G1
, G2, S and M assigned cells
phasescored_allruns <- rbind(rnaseq_seuratgenes_S,rnaseq_seuratgenes_G1,g2_m_
finalsplit)
phasescored_allruns_countmatrix <- left_join(phasescored_allruns, read_data_r
otate, by = "run")
phasescored_allruns_countmatrix <- subset(phasescored_allruns_countmatrix, se
lect = -c (orig.ident, nCount_RNA, nFeature_RNA, S.Score, G2M.Score))
phasescored_allruns_countmatrix <- data.frame(t(phasescored_allruns_countmatr
ix[]))
names(phasescored_allruns_countmatrix) <- as.matrix(phasescored_allruns_count
matrix[1, ])
phasescored_allruns_countmatrix <- phasescored_allruns_countmatrix[-1, ]

write.csv(phasescored_allruns_countmatrix, file = (paste((GSE),"All_Phases_As
signed.csv")))
```

Finally we calculate the assigned phase percentages in the complete G1, G2, S and M assigned count matrix and graph these percentages

```
#Calculates the assigned phase percentages in the overall intial count matrix
phasescored_allruns_countmatrix <- data.frame(t(phasescored_allruns_countmatr
ix[]))
phase_df1 <- phasescored_allruns_countmatrix$Phase
phase_df1 <- count(phase_df1)
names(phase_df1)[1] <- 'Phase'
sumphase <- sum(phase_df1$freq)
percentage_df <- phase_df1$freq /sumphase *100
phase_df1$Percentage <- percentage_df
phase_df1 <- phase_df1 %>%
  arrange(desc(Phase)) %>%
  mutate(prop = freq / sum(phase_df1$freq) *100) %>%
  mutate(ypos = cumsum(prop)- 0.5*prop )
percent=(phase_df1$Percentage)
percent=round(percent,digits=3)
phase_df1$PercentageLabel <- paste0((percent), "%")
```

```
#Plots as a pie chart the assigned phase percentages in the overall intial count matrix
ggplot(data = phase_df1, aes(x = "", y = Percentage, fill = Phase)) +
  geom_bar(stat = "identity") +
  coord_polar("y")  + geom_text(aes(y = ypos, label = PercentageLabel), color = "white", size=3) + scale_fill_manual(values = c("G1"= "red","S"= "darkgreen","M"= "#984DD3", "G2"="#E88A13"))
```

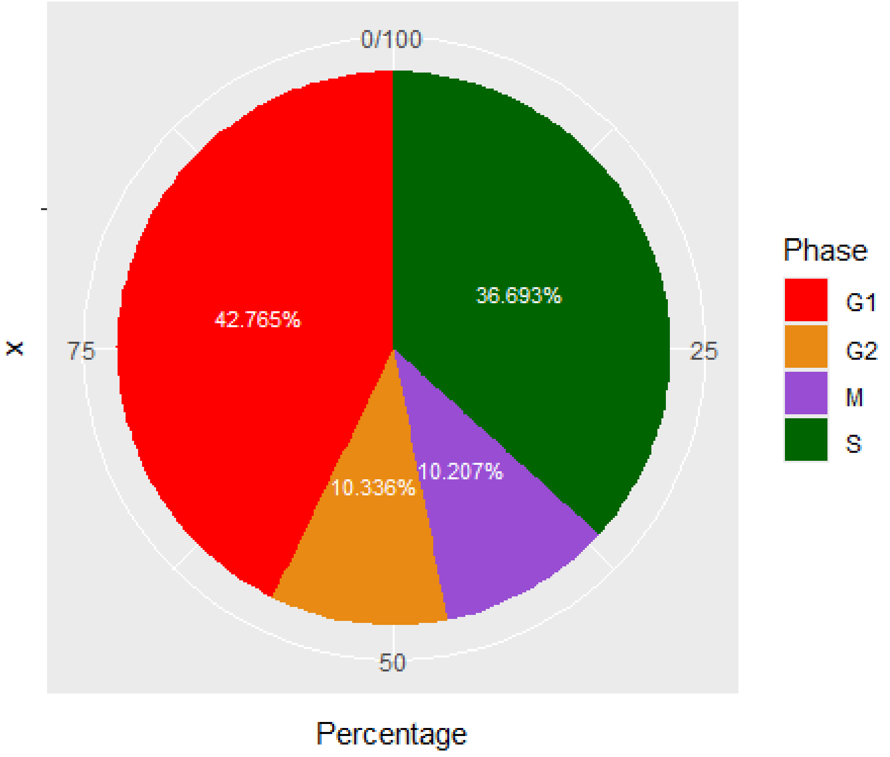

Supplement: Supplementary file 1 [file ijms-25-04589-s001.zip › ijms-2853989-supplementary.pdf]
